# The cooperative regulatory effect of the miRNA-130 family on milk fat metabolism in dairy cows

**DOI:** 10.5713/ab.23.0485

**Published:** 2024-04-25

**Authors:** Xiaofen Li, Yanni Wu, Xiaozhi Yang, Rui Gao, Qinyue Lu, Xiaoyang Lv, Zhi Chen

**Affiliations:** 1 School of Animal Science and Technology, Jiangsu Agri-animal Husbandry Vocational College, Taizhou 225300, China; 2 College of Animal Science and Technology, Yangzhou University, Yangzhou 225009, China; 3 Laboratory of Animal Developmental Biology, Department of Animal Science, Chungbuk National University, Cheongju 28644, Korea; 4 Joint International Research Laboratory of Agriculture & Agri-Product Safety, Ministry of Education, Yangzhou University, Yangzhou 225009, China; 5 International Joint Research Laboratory, Universities of Jiangsu Province of China, Domestic Animal Germplasm Resources and Genetic Improvement, Yangzhou 225009, China

**Keywords:** Dairy Cows, Milk Fat Metabolism, miR-130a, miR-130b, Peroxisome Proliferator-activated Receptor Gamma (*PPARG*)

## Abstract

**Objective:**

There is a strong relationship between the content of beneficial fatty acids in milk and milk fat metabolic activity in the mammary gland. To improve milk quality, it is therefore necessary to study fatty acid metabolism in bovine mammary gland tissue. In adipose tissue, peroxisome proliferator-activated receptor gamma (*PPARG*), the core transcription factor, regulates the fatty acid metabolism gene network and determines fatty acid deposition. However, its regulatory effects on mammary gland fatty acid metabolism during lactation have rarely been reported.

**Methods:**

Transcriptome sequencing was performed during the prelactation period and the peak lactation period to examine mRNA expression. The significant upregulation of *PPARG* drew our attention and led us to conduct further research.

**Results:**

According to bioinformatics prediction, dual-luciferase reporter system detection, real-time quantitative reverse transcription polymerase chain reaction and Western blotting, miR-130a and miR-130b could directly target *PPARG* and inhibit its expression. Furthermore, triglyceride and oil red O staining proved that miR-130a and miR-130b inhibited milk fat metabolism in bovine mammary epithelial cells (BMECs), while *PPARG* promoted this metabolism. In addition, we also found that the coexpression of miR-130a and miR-130b significantly enhanced their ability to regulate milk fat metabolism.

**Conclusion:**

In conclusion, our findings indicated that miR-130a and miR-130b could target and repress PPARG and that they also have a functional superposition effect. miR-130a and miR-130b seem to synergistically regulate lipid catabolism via the control of PPARG in BMECs. In the long-term, these findings might be helpful in developing practical means to improve high-quality milk.

## INTRODUCTION

In lactation physiology, the mammary gland is the main tissue involved in fatty acid metabolism, where a large amount of fatty acid is secreted and metabolized during lactation, thus affecting fatty acid content and milk flavor [1,2]. Although peroxisome proliferator-activated receptor gamma (*PPARG*) has been shown to be critical for fatty acid metabolism during mammary gland lactation, its specific mechanism in this process has rarely been reported [3,4]. In general, researchers believe that additives contribute to fatty acid metabolism in the mammary gland by regulating the transcription factor sterol regulatory element binding protein-1 (*SREBP-1*). In particular, *SREBP-1* has been shown to play a role in fatty acid metabolism [5,6]. Peterson et al. reported that trans-10 and cis-12 conjugated linoleic acid (CLA) affect the protein maturation of SREBP-1, which in turn affects the synthesis and content of fatty acids in bovine mammary epithelial cells (BMECs) [7]. These long-chain fatty acids are the natural ligands of *PPARG* [8]. Through (at least partially) the *PPARG-SREBP1* signaling pathway, exogenous fatty acids are likely to affect the transcription of key genes involved in the downstream lactation process during milk fat reduction [9]. *In vitro* experiments on mammary cells revealed that the addition of long-chain fatty acids can strongly increase the expression of lipid droplet formation genes, such as lipoprotein lipase (*LPL*), stearoyl-CoA desaturase 1 (*SCD1*), adipocyte differentiation-related protein (*ADFP*) and lipin1 (*LPIN1*) [10,11]. Bionaz et al [12] conducted a quantitative analysis of 54 fatty acid metabolism genes during various stages of lactation to construct a regulatory network map of different genes. Additionally, this study investigated the role of SREBP-1 and specifically emphasized the potential significance of *PPARG* in regulating fatty acid metabolism during lactation, providing a theoretical basis for our research on bovine mammary epithelial cells (BMECs) [12]. Therefore, we aimed to determine the role of the *PPARG* gene in the lactation process of dairy cows and how it interacts with microRNAs (miRNAs).

miRNAs are important noncoding RNA molecules approximately 22 nucleotides in length that are critical for degrading or inhibiting target gene translation at the posttranscriptional level [13]. Many biological and metabolic processes, such as cell proliferation, signal transduction, development, and apoptosis, are involved [14]. Dysregulation of miRNA function is therefore responsible for altering certain characteristics of cell biology, eventually causing disease and even cancer. It is also possible for miRNAs to regulate most human genes. They are thought to regulate most protein-coding genes through synergy since multiple miRNAs can work together to control a single gene. For this reason, cooperative regulation among miRNAs is essential for understanding sophisticated posttranscriptional regulation [15]. Furthermore, many complex diseases are caused by the combined action of multiple dysregulated miRNAs rather than a single miRNA. During lactation, the mammary glands of dairy cows undergo vigorous lipid metabolism [16]. Nevertheless, few studies have examined the regulatory effects of miRNAs on mammary gland fatty acid metabolism, especially the synergistic effects of miRNAs and miRNAs and between miRNAs and mRNAs.

MiR-130a and miR-130b are members of the miR-130 family [17]. Current studies suggest that the expression of miR-130 is downregulated in endometrial carcinoma, lung adenocarcinoma, and head and neck squamous cell carcinoma and that this tumor suppressor effect is mediated through TAp63 (a form of the p63 transcription factor)-regulated and epithelial–mesenchymal transition (EMT)-related mechanisms [18]. There is also evidence that miR-130a is upregulated in gastric cancer, liver cancer, and osteosarcoma [19]. In the human body, miR-130a and miR-130b are colocalized on chromosome 22 at a distance of 10 kb [20]. It is possible that these genes have similar biological functions and are expressed in tandem in BMECs. Therefore, in the present study, miR-130a/b analogs were used to transfect BMECs to study the synergistic regulatory effect of miR-130a and miR-130b on fatty acid composition, thus providing a new platform for further clarification of ruminant mammary gland milk fat synthesis and regulatory mechanisms.

## MATERIALS AND METHODS

### Ethics statement

The animal use protocol in this study was approved by the College of Biology and Oceanography, Jiangsu Agri-animal Husbandry Vocational College (2021354).

### Sample collection and RNA extraction

Three Chinese Holstein dairy cows in the prelactation period and peak lactation period were selected, and 1 to 2 g of mammary gland tissue was collected during surgery. After being washed with diethyl pyrocarbonate (DEPC; Beyotime, Beijing, China), the tissue samples were stored in liquid nitrogen. Total tissue RNA was extracted and treated according to the instructions of the relevant kit (Vazyme, Shanghai, China). Approximately 1 μg of qualified RNA was collected according to the concentration and mixed to construct an RNA pool for library construction.

### Transcriptome sequencing and analysis

After the constructed transcriptome sequencing library was analyzed via an Agilent 2100 bioanalyzer, paired-end (2×100 bp) sequencing was performed by Lianchuan Biotechnology Company using an Illumina HiSeq 2500 platform. Sample UniGenes were compared with public databases, after which functional annotation was performed according to gene similarity. Similarities between cow mammary gland tissue transcripts and genes from other species were detected (Supplementary Table S1). Moreover, the UniGenes were subjected to gene ontology (GO) and Kyoto encyclopedia of genes and genomes (KEGG) enrichment analyses to explore the potential functions of these transcripts. The above spliced genes (limited consideration to >200 bp genes) were used as a library, and sequence similarity comparison was conducted to determine the expression abundance of each gene in each sample. The software package Bowtie 0.12.8 was used to map the sequences, and the single-end mapping method was used to align the reads to multiple genes. Ultimately, the reads per kilobase of exon model per million mapped reads (RPKM) value was used to measure gene expression.

### Cell culture and small RNA transfection

For BMEC separation, we collected mammary glands from milk bovines during the peak lactation period. Then, 3× phosphate-buffered saline (PBS; Gibco, Boston, MA, USA) was used to rinse the sections, which were incubated with 3× double anti-d-Hanks solution (Gibco, USA). Next, we kept them in a lower temperature and took them back to the laboratory, there after quickly placing them in 3×PBS, cutting acinar, removing connective tissues, and cutting into pieces conserving in a small dish with 37°C for 30 min. Finally, we fetched these tissues and added 1 mL F12 medium (Gibco, USA) into them, changing the medium after 2 days. BMECs were cultured and expanded in basal culture medium supplemented with 90% Dulbecco’s modified Eagle medium (DMEM; Gibco, USA)/F12, 10% fetal bovine serum, 100 U/mL penicillin/streptomycin, 5 μg/mL insulin, 1 μg/mL hydrogenation cortisone, and 10 ng/mL growth factor (Gibco, USA). To induce the formation and secretion of lipid droplets in the BMECs 48 h before the experiment, the basal culture medium was replaced with induction medium (basal culture medium supplemented with 2 μg/mL prolactin; Gibco, USA). Small RNAs (including miRNAs and small interfering RNAs; Invitrogen, Carlsbad, CA, USA) were transfected via the reverse transfection method recommended in the instructions of Invitrogen Lipofectamine RNAiMAX (Invitrogen, USA) Reagent transfection reagent. The final concentration of small RNA was 60 Nm (Supplementary Table S2). The transfection complex was prepared with a small RNA:transfection reagent (Beyotime, China) at a ratio of 1:3. Next, the cells were incubated at room temperature for 20 min. After the cells were digested and blown into a cell suspension, 2 mL of induction medium and transfection complex were added. The cells were then quickly shaken and placed in a 37°C CO_2_ incubator for 48 h.

### Real-time quantitative reverse transcription polymerase chain reaction

Mature miRNA expression levels were determined using real-time quantitative reverse transcription polymerase chain reaction (qRT-PCR) at 95°C for 3 min, followed by 40 cycles of 95°C for 10 s and 60°C for 30 s. Total RNA (0.5 μg) was used to synthesize cDNA along with the Prime Script RT Reagent Kit (Perfect Real Time; Takara, Kyoto, Japan). The expression levels were normalized to UXT (Ubiquitously Expressed Prefoldin Like Chaperone) expression (Supplementary Table S3).

### Western blot

After the cells used for protein extraction were cultured in a 60 mm culture dish for 48 h, the medium was discarded, and the cells were washed twice with PBS in an ice bath. Next, the cells were harvested and lysed with radio immunoprecipitation assay (RIPA) buffer supplemented with 1 mM phenylmethylsulfonyl fluoride (Beyotime, China) in an ice bath, after which a cell scraper was used to scrape the cells. Next, the proteins were transferred to a 1.5 mL centrifuge tube on ice, further lysed on a shaker for 30 min, and stored at 4°C. Then, the cells were centrifuged at 12,000 rpm for 10 min, after which the total cell protein was collected. After the total cell protein concentration was determined by the bicinchoninic acid assay (Thermo, Waltham, MA, USA), protein loading buffer was added, and the samples were boiled for 10 min to denature the proteins. Total protein (20 μg) was subjected to sodium dodecyl sulfate-polyacrylamide gel electrophoresis (SDS-PAGE), and the concentrations of the proteins in the SDS-PAGE separation gel and stacking gel were 10% and 5%, respectively. After the separated total protein was transferred to the NC membrane by a semidry transfer apparatus, the membrane was blocked with 5% skim milk powder at room temperature for 1 to 2 h. After the primary antibody (Polyclonal anti-rabbit PPARA 15540–1-AP, China; and monoclonal mouse anti-β-actin; Proteintech Group, 66009–1-IG, China; polyclonal goat anti-rabbit HRP-conjugated IgG [Tiangen, Shanghai, China] was used as the secondary antibody) was added and incubated overnight at 4°C, the membrane was washed 3 to 5 times with TBST (TBS with Tween-20, 10×; Solarbio, Shanghai, China) for 5 min each. Then, the membrane was incubated with the secondary antibody at room temperature for 1 to 2 h, followed by 3 to 5 washes with TBST, and the target band was visualized with enhanced chemiluminescence (ECL; Solarbio, China) luminescence solution.

### Oil red O staining

One gram of paraformaldehyde was added to 10 mL of high-purity water, and the mixture was subsequently heated in an oven at 80°C until it was dissolved (while shaking well during this period) to prepare a 10% paraformaldehyde fixative. Oil red O staining solution was prepared by adding 0.050 g of oil red O powder (powder) to 10 mL of 70% ethanol and shaking at 37°C for at least 8 h to form a saturated solution. This solution was filtered through a 0.2 μm filter before use. The cells used for oil red O staining were washed twice with PBS and fixed with 10% paraformaldehyde at 4°C for 1 to 2 h. Then, the cells were washed with PBS again to remove paraformaldehyde and stained with oil red O (dissolved in isopropanol). The stained cells were washed twice with PBS and photographed with an inverted microscope (Beyotime, China).

### Triglyceride levels and cholesterol testing

BMECs were cultured in 6-well culture plates, and each treatment was replicated 3 times. After 48 h, triglyceride (TAG) and cholesterol (Loogen, Shanghai, China) levels were determined. The cells were then washed with PBS and lysed in lysis buffer. Thereafter, the samples were collected into a 1.5 mL centrifuge tube and incubated at 4°C for 15 min before centrifugation was used to collect the supernatant and measure its content. Protein quantification was performed on the supernatant (10 μL) using a protein assay kit (Thermo, USA). The supernatant was heated at 70°C for 10 min and centrifuged at 2,000 rpm for 5 min. The triglyceride and cholesterol contents in the supernatant were determined via an enzymatic method (Abcam, Boston, MA, USA). A standard curve was drawn to calculate TAG and cholesterol concentrations.

### Luciferase activity assay

The miRNA target gene online analysis software packages TargetScan (https://www.targetscan.org/) and PicTar (https://pictar.mdc-berlin.de/) were used to predict and comprehensively analyze the miRNAs targeted by *PPARG*. The results revealed that miR-130a/miR-130b could target the *PPARG* gene. The *PPARG* gene 3′-UTR was used as a template for designing and amplifying the miR-130a/miR-130b interaction sites, and *Xho* I and *Not* I restriction sites were also added. The 3′-UTR fragment of the *PPARG* gene amplified by PCR and the psiCHECK-2 luciferase vector were both digested with *Xho* I and *Not* I (Supplementary Table S4). Following overnight incubation at 4°C under the action of T4 ligase, the proteins were prepared for use in the next step. After the BMECs had been seeded in 48-well Plates a day prior to transfection, the cells were transfected once the density of the cells reached approximately 50,000 per well (Vazyme, China).

### Statistical analysis

The different treatment groups were analyzed using one-way analysis of variance and SPSS (Statistical Program for Social Sciences) 19.0, and the results are expressed as the mean± standard deviation. * p<0.05 indicates a significant difference, and ** p<0.01 indicates an extremely significant difference.

## RESULTS

### Transcriptome analysis of mammary gland tissue of dairy cows in different lactation stages

**Figure 1:**
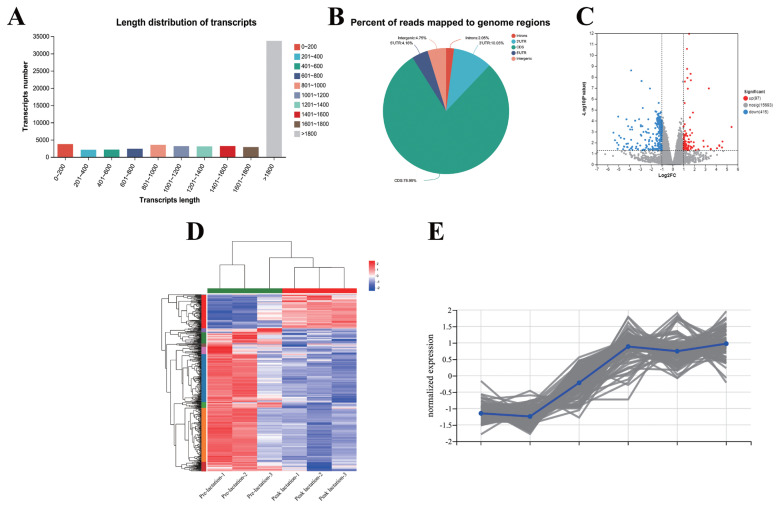
Transcriptome sequencing of breast tissues from the prelactation period and the peak lactation period. (A) Transcript length distribution. Sample UniGenes were compared with public databases, after which functional annotation was performed according to gene similarity. (B) Statistics on the distribution of reads in the prelactation period and the peak lactation period. Based on the analysis results, the distribution of reads was determined using the genomes in different regions of the reference genome as a benchmark. The positioning regions were divided into coding regions (CDSs), introns, intergenic regions and UTRs (5′ and 3′ untranslated regions), and most of the reads were distributed in CDSs. (C and D) Volcano plot of the expression differences and Cluster analysis of the preo-lactation period and the peak lactation period. Gene expression analysis revealed 97 differentially expressed genes upregulated and 415 differentially expressed genes downregulated. (E) Subcluster trend chart. A clustering analysis of the expression patterns of the genes/transcripts in the selected gene sets.

**Figure 2:**
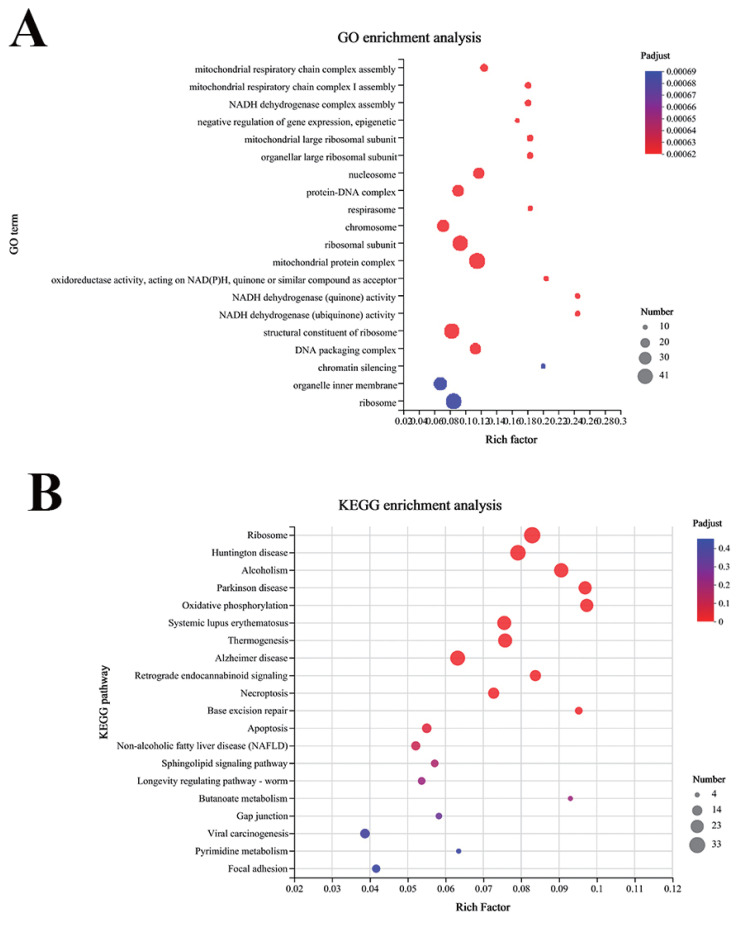
GO enrichment analysis and KEGG enrichment analysis. The UniGenes were subjected to gene ontology (GO) and Kyoto encyclopedia of genes and genomes (KEGG) enrichment analyses to explore the potential functions of these transcripts. The above spliced geneswere used as a library, and sequence similarity comparison was conducted to determine the expression abundance of each gene in each sample. (A) GO enrichment analysis: The top 30 differentially expressed genes (DEGs) were grouped into different categories: mitochondrial respiratory chain complex assembly, NADH dehydrogenase complex assembly, negative regulation of gene expression, epigenetic, and organellar large ribosomal subunit. (B) KEGG enrichment analysis: 61 pathways were enriched, including ribosome, oxidative phosphorylation, retrograde endocannabinoid signaling, apoptosis, and nonalcoholic fatty liver disease (NAFLD).

Transcriptome sequencing of mammary gland tissues from dairy cows throughout the prelactation period and the peak lactation period was performed. Based on the analysis results, the distribution of reads was determined using the genomes in different regions of the reference genome as a benchmark. The positioning regions were divided into coding regions (CDSs), introns, intergenic regions and UTRs (5′ and 3′ untranslated regions), and most of the reads were distributed in CDSs (Figure 1A, B). Furthermore, gene expression analysis revealed 97 differentially expressed genes upregulated (including PPARG) and 415 differentially expressed genes downregulated (Figure 1C, 1D). Thereafter, we performed a clustering analysis of the expression patterns of the genes/transcripts in the selected gene sets (Figure 1E). The genes obtained from these regions were used as potential research objects. To gain a deeper understanding of the transcriptional regulatory relationship between miRNAs and their parental genes, we performed GO enrichment analysis and Kyoto encyclopedia of genes and genomes (KEGG) pathway enrichment analysis on the DEGs. According to the GO analysis, the top 30 DEGs were grouped into different categories: mitochondrial respiratory chain complex assembly, NADH dehydrogenase complex assembly, negative regulation of gene expression, epigenetic, and organellar large ribosomal subunit (Figure 2A). According to the KEGG results, 61 pathways were enriched, including ribosome, oxidative phosphorylation, retrograde endocannabinoid signaling, apoptosis, and nonalcoholic fatty liver disease (NAFLD) (Figure 2B). It is therefore concluded that miRNAs produced by these genes may exert effects on the body through these pathways.

### Transfection efficiency of miRNA and siRNA

**Figure 3:**
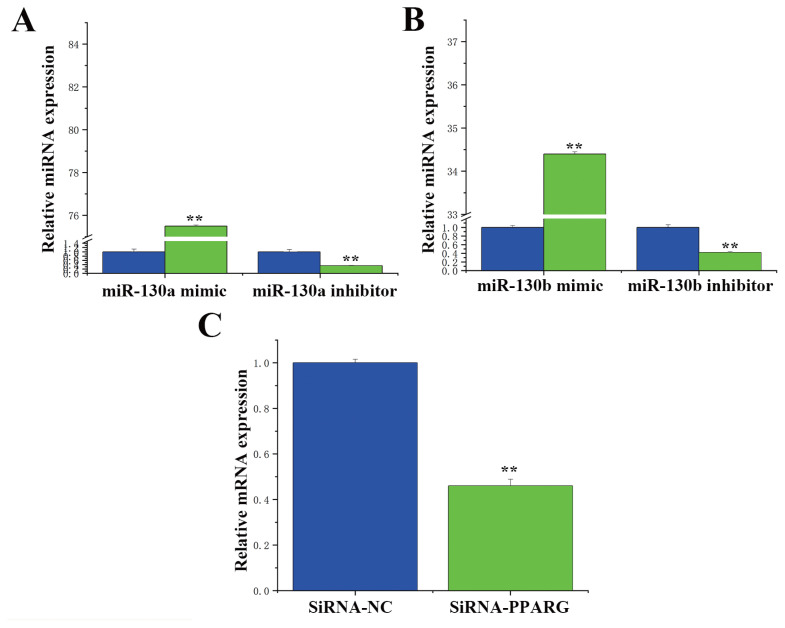
The transfection efficiency of miR-130a, miR-130b, and siRNA-peroxisome proliferator-activated receptor gamma (*PPARG*). (A) Expression levels of miR-130a. Real-time quantitative reverse transcription polymerase chain reaction (qRT-PCR) quantification of miR-130a expression (n = 6). The expression levels of the miR-130a mimic was 75 times greater, 70% of the genes was downregulated in the miR-130a inhibitor groups. The blue bars represent the negative control; the green bars represent the miR-130a mimic or inhibitor. (B) Expression levels of miR-130b. qRT-PCR quantification of miR-130b expression (n = 6). The expression levels of the miR-130b mimic was 34 times greater, 62% of the genes was downregulated in the miR-130b inhibitor groups. The blue bars represent the negative control; the green bars represent the miR-130b mimic or inhibitor. (C) mRNA expression levels of *PPARG*. qRT-PCR quantification of *PPARG* expression (n = 6). The blue bars represent the siRNA-NCs; the green bars represent the siRNA-*PPARγ*. The expression levels of siRNA-PPARG were downregulated by nearly 60%. The values are the means±standard error of the means. * p<0.05; ** p<0.01.

qRT-PCR was used to verify the transfection efficiency of the miR-130a mimic and inhibitor in BMECs. As shown in Figure 3A and 3B, compared with those in the control group (NC-mimic), the expression levels of the miR-130a mimic and miR-130b mimic were 75 and 34 times greater, respectively, after treatment. In contrast, 70% and 62% of the genes were downregulated in the miR-130a inhibitor and miR-130b inhibitor groups, respectively, compared with the control group (NC-Inhibitor). These results indicate that the miR-130a mimic and inhibitor exhibit increased transfection efficiency and can be used in subsequent experiments, as can the miR-130b mimic and inhibitor. Then, we used qRT-PCR and Western blotting to verify the transfection efficiency of siRNA-*PPARG* in the BMECs. Figure 3C shows that, compared with those in the siRNA-NC group, the expression levels of siRNA-*PPARG* were downregulated by nearly 60%. Hence, siRNA can be used in subsequent experiments because of its high transfection efficiency.

### MiR-130a and miR-130b each specifically target *PPARG* in BMECs

**Figure 4:**
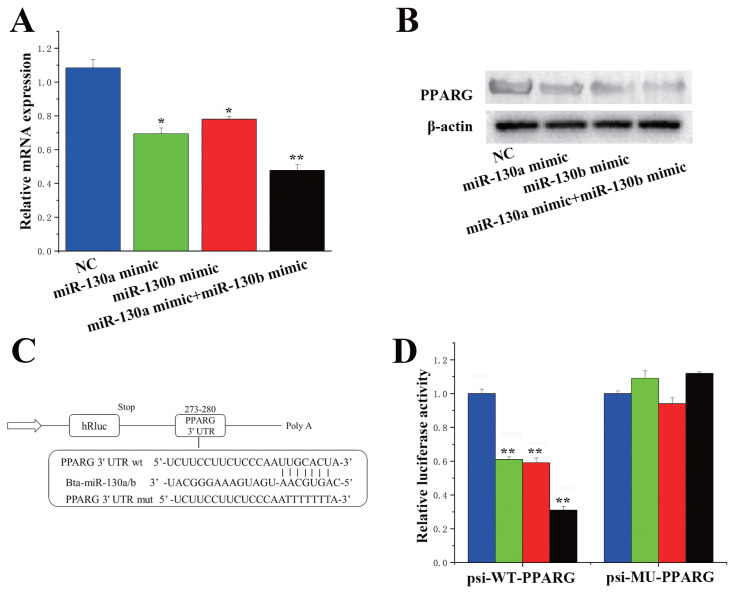
MiR-130a/b specifically targets peroxisome proliferator-activated receptor gamma (*PPARG*). (A) Expression levels of *PPARG* in the miR-130a/b mimic and miR-130a mimic+miR-130b mimic groups. The blue bars represent the negative control, the green bars represent the miR-130a mimic, the red bars represent the miR-130b mimic, and the black bars represent the miR-130a mimic and miR-130b mimic. miR-130a mimic downregulated the mRNA expression of the *PPARG* gene, and the miR-130b mimic also had the same effect on the *PPARG* gene. (B) The protein expression level of *PPARG*. The protein expression level of PPARG was consistent with the mRNA expression level. (C) Target sites of miR-130a/b in the PPARG 3′-UTR. The 3′-UTR of the PPARG gene was combined with the miR-130a/b site. (D) Construction of the luciferase (Luc) vector fused with the *PPARG* 3′-UTR. WT, Luc reporter vector with wild-type *PPARG* 3′-UTR; MU, Luc reporter vector with the mutation at the miR-130a/b site in the *PPARG* 3′-UTR. The 3′-UTR activity of the wild-type *PPARG* gene was inhibited by miR-130a/b overexpression, while its activity was not affected in the mutants. The blue bars represent the negative control, the green bars represent the miR-130a mimic, the red bars represent the miR-130b mimic, and the black bars represent the miR-130a mimic and miR-130b mimic. * p<0.05, ** p<0.01.

To select *PPARG* as the research object, it is necessary to determine the relationships between miR-130a and miR-130b and their target genes. Using the online software TargetScan 6.2 and the miRNA function analysis software DAVID (https://david.ncifcrf.gov/summary), on the premise that the miRNA and the *PPARG* 3′-UTR were completely matched, many miR-130a and miR-130b were predicted. One miRNA was a *PPARG* target gene. We found that the miR-130a mimic downregulated the mRNA expression of the *PPARG* gene, and the miR-130b mimic also had the same effect on the *PPARG* gene (Figure 4A). Similarly, in the presence of both miR-130a and miR-130b, the protein expression level of *PPARG* was consistent with the mRNA expression level (Figure 4B). Notably, when miR-130a and miR-130b were overexpressed simultaneously in BMECs, we observed greater downregulation of *PPARG* mRNA and protein expression than when they were overexpressed alone. These findings indicate that there may be a synergistic relationship between miR-130a and miR-130b. On the other hand, as shown in Figure 4C, the 3′-UTR of the *PPARG* gene was combined with the miR-130a/b site. To determine whether miR-130a/b can directly target these sites, we synthesized a *PPARG* 3′-UTR fragment, which contains the target site of miR-130a/b. This fragment was subsequently transferred to the psi-CHECK2 vector and cloned for identification. According to the luciferase reporter assay, the 3′-UTR activity of the wild-type *PPARG* gene was inhibited by miR-130a/b overexpression, while its activity was not affected in the mutants (Figure 4D).

### The functions of miR-130a and miR-130b in bovine mammary epithelial cells

**Figure 5:**
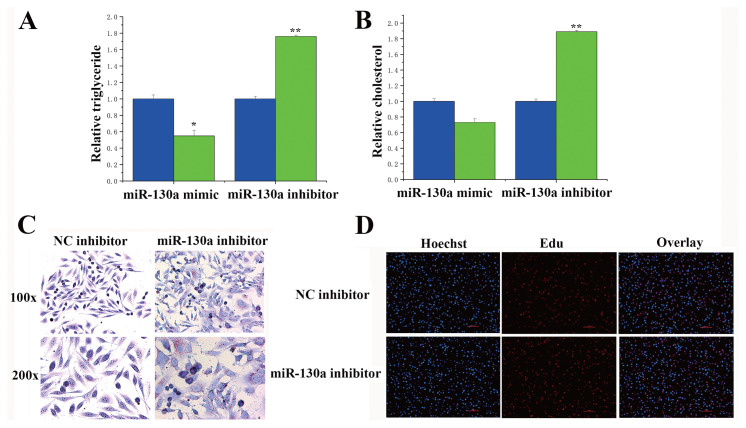
Functional evaluation of miR-130a. (A) Relative triglyceride levels. The content of TAG was markedly lower in the miR-130a mimic treatment group. The values are the means± standard error of the means. * p<0.05; ** p<0.01. The blue bars represent the negative control; the green bars represent the miR-130a mimic or inhibitor. (B) Relative cholesterol levels. The blue bars represent the negative control, and the green bars represent the miR-130a mimic or inhibitor. The miR-130a inhibitor substantially upregulated the cholesterol concentration. The values are the means±standard error of the means. ** p<0.01. The blue bars represent the negative control; the green bars represent the miR-130a mimic or inhibitor. (C) Oil red O staining. The miR-130a inhibitor treatment group exhibited more lipid droplets than did the NC inhibitor group. (D) Results from the EdU experiment of BMECs in the NC inhibitor and miR-130a inhibitor groups. The miR-130a inhibitor did not affect the proliferation of the BMECs.

**Figure 6:**
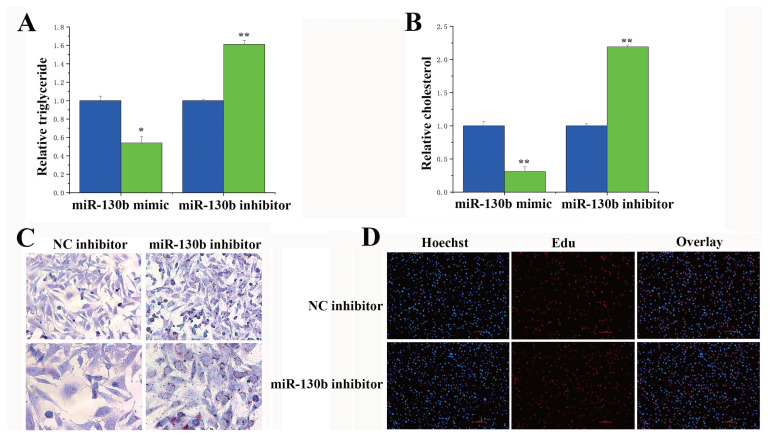
Functional evaluation of miR-130b. (A) Relative triglyceride levels. The content of TAG was markedly lower in the miR-130b mimic treatment group. The values are the means±standard error of the means. * p<0.05; ** p<0.01. The blue bars represent the negative control; the green bars represent the miR-130a mimic or inhibitor. (B) Relative cholesterol levels. The blue bars represent the negative control, and the green bars represent the miR-130b mimic or inhibitor. The miR-130b inhibitor substantially upregulated the cholesterol concentration. The values are the means± standard error of the means. ** p<0.01. The blue bars represent the negative control; the green bars represent the miR-130a mimic or inhibitor. (C) Oil red O staining. The miR-130b inhibitor treatment group exhibited more lipid droplets than did the NC inhibitor group. (D) Results from the EdU experiment of the bovine mammary epithelial cells (BMECs) in the NC inhibitor and miR-130b inhibitor groups. The miR-130b inhibitor did not affect the proliferation of the BMECs.

The milk fat in BMECs consists of many small milk fat droplets derived from triglycerides. Under conditions of overexpression and inhibition of miR-130a/b, we detected changes in the number of milk fat droplets and in the TAG content in the BMECs. As shown in Figure 5A, compared with those in the control group, the content of TAG was markedly lower in the miR-130a mimic treatment group, whereas the content was upregulated in the miR-130a inhibitor treatment group. Similarly, the miR-130a inhibitor substantially upregulated the cholesterol concentration (Figure 5B). According to the morphological observation of oil red O staining, the miR-130a inhibitor treatment group exhibited more lipid droplets than did the NC inhibitor group (Figure 5C). EdU results revealed that the miR-130a inhibitor did not affect the proliferation of the BMECs (Figure 5D). The above experiments prove that miR-130a regulates milk fat metabolism in BMECs, and the same result can be found for miR-130b (Figure 6A–D).

### The function of peroxisome proliferator-activated receptor gamma in bovine mammary epithelial cells

**Figure 7:**
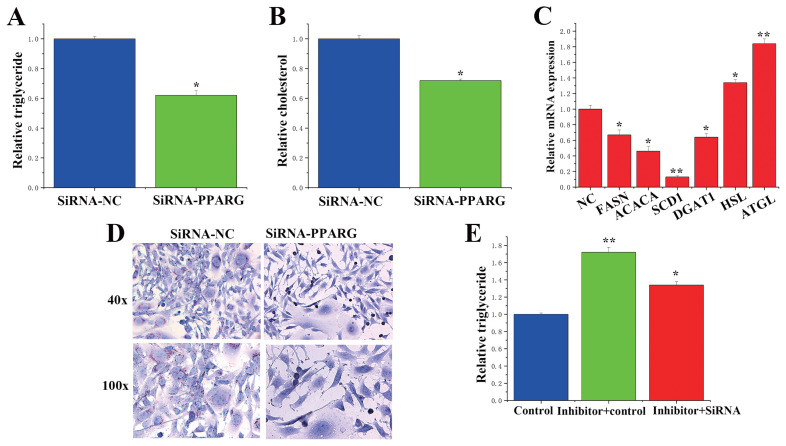
Functional evaluation of peroxisome proliferator-activated receptor gamma (*PPARG*). (A) Relative triglyceride levels. The content of triglyceride was markedly lower in the siRNA-*PPARG*. The values are the means±standard error of the means. * p<0.05; ** p<0.01. (B) Relative cholesterol levels. The content of cholesterol was markedly lower in the siRNA-*PPARG*. The values are the means±standard error of the means. * p<0.05; ** p<0.01. (C) Effect of siRNA-*PPARG* on the mRNA abundance of genes related to lactation. The expression levels of FASN, ACACA, SCD1 and DGAT1 were significantly downregulated, while those of HSL and ATGL were drastically upregulated. (D) Oil red O staining. The lipid droplet content was markedly lower in the siRNA-*PPARG*. (E) The expression of PPARG in the control, miR-130a inhibitor + miR-130a inhibitor, and miR-130a inhibitor + miR-130a inhibitor + siRNA-*PPARG* groups. Inhibition of miR-130a and miR-130b expression could dramatically increase TAG expression levels. The blue bars represent the negative control; the green bars represent the miR-130a inhibitor + miR-130a inhibitor, red bars represent the miR-130a inhibitor + miR-130a inhibitor + siRNA-*PPARG* groups. * p<0.05, ** p<0.01.

To verify the function of *PPARG* in BMECs, we treated *PPARG* with small interfering RNA (siRNA) and conducted follow-up studies. In siRNA-*PPARG*-treated BMECs, TAG and cholesterol levels were significantly lower than those in siRNA-NC-treated cells (Figure 7A, B), and the lipid droplet content was also considerably lower according to the oil red O staining results (Figure 7D). Additionally, in terms of the expression of genes related to milk fat metabolism, the expression levels of fatty acid synthase (*FASN*), acetyl-CoA carboxylase alpha (*ACACA*), *SCD1*, and diacylglycerol acyltransferase (*DGAT1*) were significantly downregulated, while those of hormone-sensitive lipase (*HSL*) and adipose triacylglyceride lipase (ATGL) were drastically upregulated (Figure 7C). As a result, *PPARG* appears to promote milk fat metabolism in BMECs.

### Synergistic effects of miR-130a and miR-130b in enhancing the regulation of milk fat metabolism

**Figure 8:**
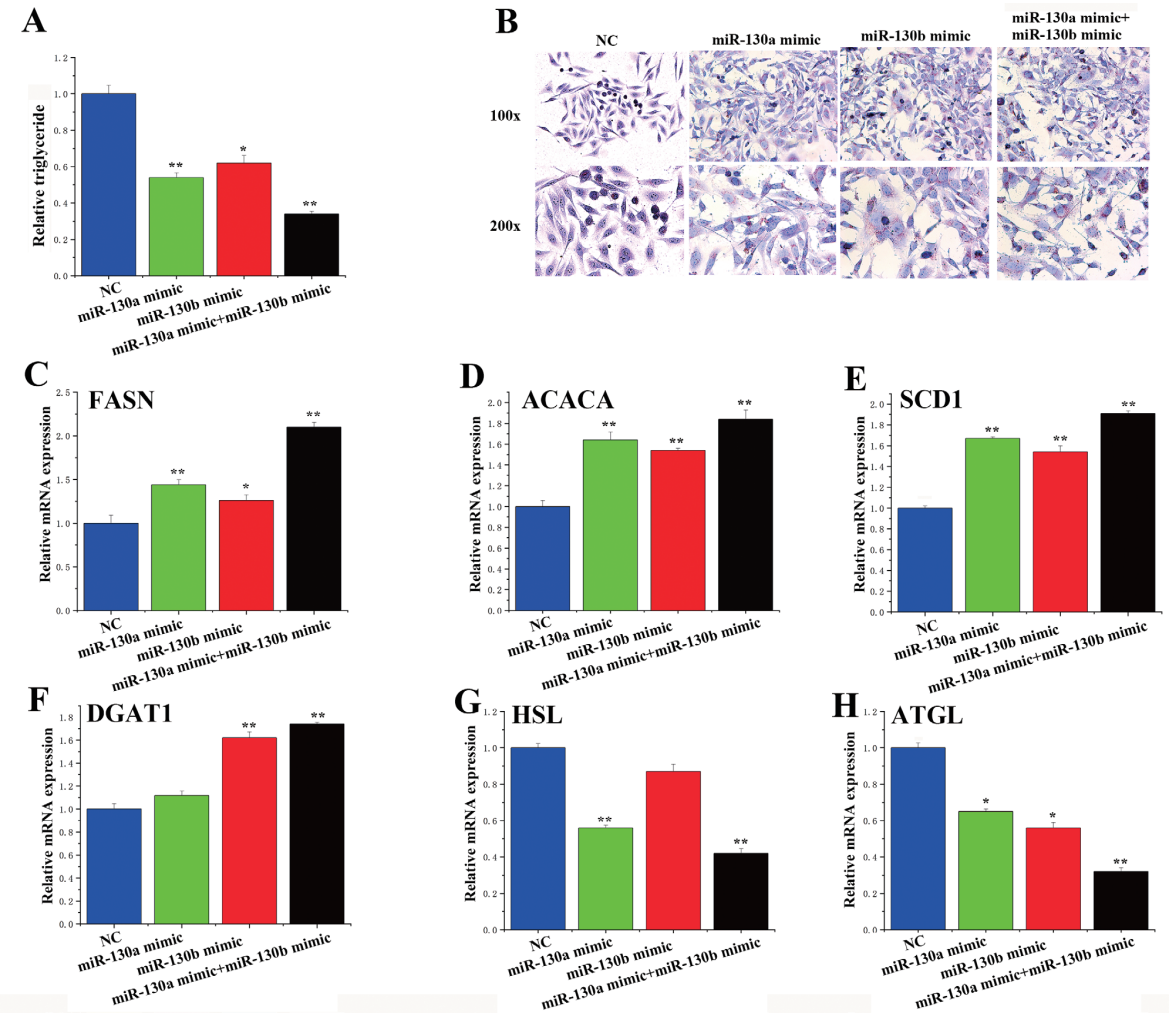
MiR-130a and miR-130b cooperate to inhibit peroxisome proliferator-activated receptor gamma (*PPARG*). (A) Relative triglyceride levels. The overexpression of miR-130a/b upregulated TAG expression. (B) Oil red O staining. Bovine mammary epithelial cells (BMECs) presented a substantial increase in lipid droplet content when both miR-130a and miR-130b were inhibited. The relative mRNA expression of (C) *FASN*, (D) *ACACA*, (E) *SCD1*, (F) *DGAT1*, (G) *HSL*, and (H) *ATGL*. The blue bars represent the negative control, the green bars represent the miR-130a mimic, the red bars represent the miR-130b mimic, and the black bars represent the miR-130a mimic and miR-130b mimic. All the experiments were performed in duplicate and repeated three times. Overexpression of miR-130a and miR-130b significantly upregulated the expression levels of FASN, ACACA, SCD1 and DGAT1 but downregulated the expression levels of HSL and ATGL. * p<0.05, ** p<0.01.

As previously observed, there was synergistic enhancement of the regulatory effect of miR-130a and miR-130b (Figure 4A); therefore, we wondered whether this effect also occurred during milk fat metabolism. Therefore, we overexpressed miR-130a and miR-130b in the BMECs simultaneously. The TAG assay results showed that, as before, the overexpression of miR-130a/b upregulated TAG expression. In contrast, the combined overexpression of miR-130a and miR-130b downregulated TAG expression (Figure 8A). In terms of genes related to milk fat metabolism, combined overexpression of miR-130a and miR-130b significantly upregulated the expression levels of *FASN*, *ACACA*, *SCD1*, and *DGAT1* (Figure 8C–F) but downregulated the expression levels of *HSL* and *ATGL* (Figure 8G, H). Notably, BMECs presented a substantial increase in lipid droplet content when both miR-130a and miR-130b were inhibited (Figure 8B). In this manner, we were able to determine the synergistic effect of miR-130a and miR-130b.

### miR-130a and miR-130b participate in milk fat metabolism by regulating peroxisome proliferator-activated receptor gamma

Previous experiments have shown that miR-130a and miR-130b inhibit TAG synthesis, while their target gene *PPARG* promotes this synthesis. To investigate the relationship between miR-130a/b and *PPARG*, we performed a “rescue” experiment in BMECs and revealed that inhibition of miR-130a and miR-130b expression could dramatically increase TAG expression levels (Figure 7E). In contrast, the inhibition of *PPARG* prevented the increase in TAG caused by siRNA-*PPARG* rescue, demonstrating that miR-130a/b functions through *PPARG*.

## DISCUSSION

### The miR-130 family (miR-130a/b) is the main research object

Since the discovery of the first miRNA gene in the study of nematode development by Lee and Dutta [21] in 2009, a growing number of miRNAs have been discovered, and their functions and roles in regulating gene expression have also received increasing attention. To date, more than 48,000 miRNAs, including 2,693 human-derived miRNAs, have been identified [22,23]. It is predicted that miRNAs directly regulate more than half of human genes since a single miRNA can target up to 400 different mRNAs. Furthermore, abnormal expression of miRNAs has been revealed to be a feature of many pathological processes, including cancer, metabolic disorders, inflammation, and cardiovascular, neurodevelopmental, and autoimmune diseases [21,24,25]. With more in-depth research on miRNAs, researchers have screened a large number of miRNAs related to milk fat metabolism regulation and conducted in-depth and systematic functional verification and mechanism of action research on some of the screened miRNAs. The results indicate that miR-183, miR-30e-5, miR-15a, and miR-27a are important regulatory factors for breast tissue fat metabolism and play important regulatory roles in breast lactation and milk fat metabolism processes [26–28]. Although a large number of differentially expressed miRNAs in breast tissue have been identified through high-throughput sequencing technology and that some candidate miRNAs have been functionally validated and studied for their mechanisms of action, the regulatory mechanisms of a large number of candidate miRNAs (such as miR-130a and miR-130b) are still unclear. Therefore, more in-depth molecular mechanism research is needed.

### miR-130 family members synergistically regulate mammary gland milk fat metabolism

In the maturation process, miRNAs go through two forms, pri-miRNAs and pre-miRNAs [29]. Through complementary base pairing with target gene mRNAs, they can induce mRNA degradation or translation inhibition, thereby regulating genes and participating in the biological activities of the midbody. Mimics can be effectively used to screen miRNAs that regulate gene expression and affect cell development by simulating the activity of microRNAs *in vivo*, thereby helping researchers to examine gain-of function effects and discover and verify target genes of endogenous miRNAs [30]. To study whether miR-130 family members synergistically regulate mammary gland milk fat metabolism, we independently and simultaneously transfected miR-130a and miR-130b mimics into BMECs. Changes in the expression of genes related to lipid metabolism and the accumulation of lipid droplets and TAG were monitored. A significant reduction in lipid droplets and TAG levels was subsequently observed when mimic-mediated miR-130a and miR-130b were intracellularly overexpressed. Triglyceride synthesis was also found to be strongly affected by miR-130a and miR-130b. When miR-130a and miR-130b were transferred into cells together, the effect was greater than when they were transferred separately, indicating a synergistic regulatory effect. In addition, mimics of the miR-130 family were used to study the cooperative regulatory effect of miR-130a and miR-130b on mammary gland fatty acid metabolism. Cotransfected cells showed differences in lipid metabolism-related genes and lipid droplet changes compared with those in cells transfected with the other agents.

### Peroxisome proliferator-activated receptor gamma is closely related to milk fat metabolism

There has been extensive and intensive research on the effect of *PPARG* on fatty acid metabolism in nonruminant adipocytes [31,32]. However, the function of *PPARG* in breast tissue has rarely been studied. In dairy cow mammary gland tissue, activation of *PPARG* can promote the upregulation of fatty acid metabolism genes [33]. These findings revealed that *PPARG* also plays a critical role in the mammary gland tissue of dairy cows. At present, we have cloned two subtypes of *PPARG* from dairy cow mammary gland tissue. *PPARG* in BMECs was first inhibited to refine the function of *PPARG* in the regulation of mammary gland fatty acid metabolism and clarify differences among multiple subtypes. Our research revealed that *PPARG* can widely regulate the expression of genes related to fatty acid metabolism, including *FASN*, *ACACA*, *SCD1*, *DGAT1*, *HSL*, and *ATGL*. Bionaz and Loor [34,35] reported that in *in vitro* experiments on breast cells, the addition of long-chain fatty acids significantly increased the expression levels of genes involved in lipid droplet formation, such as *LPL*, *SCD*, *ADFP*, and *LPIN1* [34,35]. Our research is consistent with that of Bionaz and Loor [34,35].

In addition, *PPARG* was revealed to be the predominant form of GEL in breast tissue. It is therefore speculated that this gene is a very important regulatory factor in the lactation process that affects the network of fatty acid metabolism. This study, however, has not fully elucidated how *PPARG* affects the fatty acid composition in cells, and further studies are essential for elucidating and confirming its mechanism. This study focused on PPARG and investigated its main functions during lactation, elucidating the lipid metabolism network related to PPARG during lactation in cows and providing a theoretical basis for future lactation regulation.

## CONCLUSION

**Figure 9:**
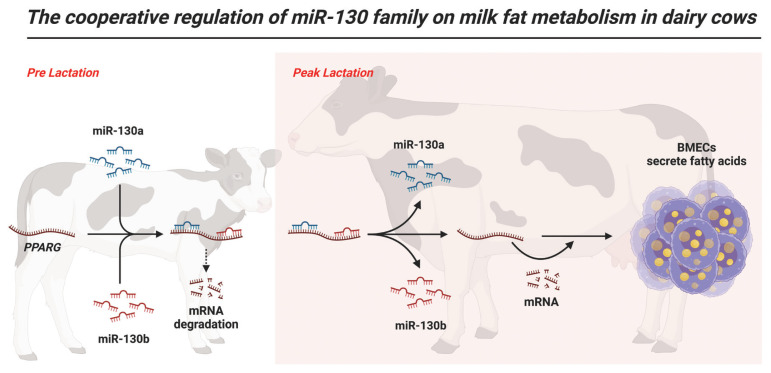
The cooperative regulatory effect of the miR-130 family on milk fat metabolism in dairy cows. miR-130a and miR-130b could target and repress peroxisome proliferator-activated receptor gamma (PPARG) and that they also have a functional superposition effect. miR-130a and miR-130b seem to synergistically regulate lipid catabolism via the control of PPARG in bovine mammary epithelial cells (BMECs).

In general, we not only identified the miR-130 family by comparing the differential expression of miRNAs at different lactation stages but also found that miR-130a and miR-130b can participate in milk fat metabolism through the coordinated regulation of the *PPARG* gene (Figure 9).

## Data Availability

Data in the manuscript was unpublished. The datasets analyzed during the current study are available from the corresponding author on reasonable request. If the article is accepted for publication, the data availability statement will be published as part of the accepted article.

## SUPPLEMENTARY MATERIAL

**Supplementary Table S1**. Transcriptome sequencing and analysis

**Supplementary Table S2**. Small RNAsequence **Supplementary Table S3**. Gene primers **Supplementary Table S4**. Luciferase vector primers

## References

[1] Chen Z, Lu Q, Zhang X (2022). Circ007071 Inhibits unsaturated fatty acid synthesis by Interacting with miR-103-5p to enhance PPARγ expression in the dairy goat mammary gland. J Agric Food Chem.

[2] Chen Z, Cao X, Lu Q (2021). circ01592 regulates unsaturated fatty acid metabolism through adsorbing miR-218 in bovine mammary epithelial cells. Food Funct.

[3] Wang MQ, Zhou CH, Cong S (2021). Lipopolysaccharide inhibits triglyceride synthesis in dairy cow mammary epithelial cells by upregulating miR-27a-3p, which targets the PPARG gene. J Dairy Sci.

[4] Wilson HE, Stanton DA, Rellick S, Geldenhuys W, Pistilli EE (2021). Breast cancer-associated skeletal muscle mitochondrial dysfunction and lipid accumulation is reversed by PPARG. Am J Physiol Cell Physiol.

[5] Pham DV, Tilija Pun N, Park PH (2021). Autophagy activation and SREBP-1 induction contribute to fatty acid metabolic reprogramming by leptin in breast cancer cells. Mol Oncol.

[6] Zhao Q, Lin X, Wang G (2022). Targeting SREBP-1-mediated lipogenesis as potential strategies for cancer. Front Oncol.

[7] Peterson DG, Matitashvili EA, Bauman DE (2004). The inhibitory effect of trans-10, cis-12 CLA on lipid synthesis in bovine mammary epithelial cells involves reduced proteolytic activation of the transcription factor SREBP-1. J Nutr.

[8] Lu Q, Zong W, Zhang M, Chen Z, Yang Z (2022). The overlooked transformation mechanisms of VLCFAs: peroxisomal β-oxidation. Agriculture.

[9] Chen GH, Luo Z, Hogstrand C, Wu K, Ling SC (2018). SREBP1, PPARG and AMPK pathways mediated the Cu-induced change in intestinal lipogenesis and lipid transport of yellow catfish Pelteobagrus fulvidraco. Food Chem.

[10] Mu T, Hu H, Ma Y, Feng X, Zhang J, Gu Y (2021). Regulation of key genes for milk fat synthesis in ruminants. Front Nutr.

[11] Zaidi N, Lupien L, Kuemmerle NB, Kinlaw WB, Swinnen JV, Smans K (2013). Lipogenesis and lipolysis: the pathways exploited by the cancer cells to acquire fatty acids. Prog Lipid Res.

[12] Bionaz M, Loor JJ (2011). Gene networks driving bovine mammary protein synthesis during the lactation cycle. Bioinform Biol Insights.

[13] Chen Z, Chu S, Liang Y (2020). miR-497 regulates fatty acid synthesis via LATS2 in bovine mammary epithelial cells. Food Funct.

[14] Sempere LF, Azmi AS, Moore A (2021). microRNA-based diagnostic and therapeutic applications in cancer medicine. Wiley Interdiscip Rev RNA.

[15] Lu Q, Chen Z, Ji D (2021). Progress on the regulation of ruminant milk fat by noncoding RNAs and ceRNAs. Front Genet.

[16] Chen Z, Lu Q, Liang Y (2021). Circ11103 interacts with miR-128/PPARGC1A to regulate milk fat metabolism in dairy cows. J Agric Food Chem.

[17] Monoe Y, Jingushi K, Kawase A (2021). Pharmacological inhibition of miR-130 family suppresses bladder tumor growth by targeting various oncogenic pathways via PTPN1. Int J Mol Sci.

[18] Zhang HD, Jiang LH, Sun DW, Li J, Ji ZL (2017). The role of miR-130a in cancer. Breast Cancer.

[19] Wei MC, Wang YM, Wang DW (2021). miR-130a-mediated KLF3 can inhibit the growth of lung cancer cells. Cancer Manag Res.

[20] Egawa H, Jingushi K, Hirono T (2016). The miR-130 family promotes cell migration and invasion in bladder cancer through FAK and Akt phosphorylation by regulating PTEN. Sci Rep.

[21] Nisenblat V, Bossuyt PM, Shaikh R (2016). Blood biomarkers for the non-invasive diagnosis of endometriosis. Cochrane Database Syst Rev.

[22] Zhou Q, Liu J, Quan J, Liu W, Tan H, Li W (2018). MicroRNAs as potential biomarkers for the diagnosis of glioma: a systematic review and meta-analysis. Cancer Sci.

[23] Kamity R, Sharma S, Hanna N (2019). MicroRNA-mediated control of inflammation and tolerance in pregnancy. Front Immunol.

[24] Wang L, Sinnott-Armstrong N, Wagschal A (2020). A microRNA linking human positive selection and metabolic disorders. Cell.

[25] Lee YS, Dutta A (2009). MicroRNAs in cancer. Annu Rev Pathol.

[26] Chen Z, Shi H, Sun S (2018). MiR-183 regulates milk fat metabolism via MST1 in goat mammary epithelial cells. Gene.

[27] Chen Z, Qiu H, Ma L (2016). miR-30e-5p and miR-15a synergistically regulate fatty acid metabolism in goat mammary epithelial cells via LRP6 and YAP1. Int J Mol Sci.

[28] Tang KQ, Wang YN, Zan LS, Yang WC (2017). miR-27a controls triacylglycerol synthesis in bovine mammary epithelial cells by targeting peroxisome proliferator-activated receptor gamma. J Dairy Sci.

[29] Alarcón CR, Lee H, Goodarzi H, Halberg N, Tavazoie SF (2015). N6-methyladenosine marks primary microRNAs for processing. Nature.

[30] Lu TX, Rothenberg ME (2018). MicroRNA. J Allergy Clin Immunol.

[31] Ma S, Zhou B, Yang Q (2021). A transcriptional regulatory loop of master regulator transcription factors, PPARG, and fatty acid synthesis promotes esophageal adenocarcinoma. Cancer Res.

[32] Zheng JS, Chen J, Wang L (2018). Replication of a gene-diet interaction at CD36, NOS3 and PPARG in response to Omega-3 fatty acid supplements on blood lipids: a double-blind randomized controlled trial. EBioMedicine.

[33] Jing Y, Chen Y, Wang S (2021). Circadian gene PER2 silencing downregulates PPARG and SREBF1 and suppresses lipid synthesis in bovine mammary epithelial cells. Biology.

[34] Bionaz M, Loor JJ (2008). Gene networks driving bovine milk fat synthesis during the lactation cycle. BMC Genomics.

[35] Bionaz M, Loor JJ (2007). Identification of reference genes for quantitative real-time PCR in the bovine mammary gland during the lactation cycle. Physiol Genomics.

