# Supplementary material for: The cooperative regulatory effect of the miRNA-130 family on milk fat metabolism in dairy cows
Source: Anim Biosci. 2024 Apr 25;37(7):1289–302. doi: 10.5713/ab.23.0485 (PMC11222843; doi:10.5713/ab.23.0485)
Supplement: Supplementary file 1 [file ab-23-0485-Supplementary-Table-1.pdf]

Supplementary table S1: Transcriptome sequencing and analysis

Gene description	FC(Experience/NC)	Log2FC(Experience/NC)	Pvalue
ATP synthase F0 subunit 8 [Source:NCBI gene (formerly Entrezgene);Acc:3283881]	2.861	1.516666	1.10E-12
NADH dehydrogenase subunit 1 [Source:NCBI gene (formerly Entrezgene);Acc:3283877]	2.49	1.316256	2.66E-11
ATP synthase F0 subunit 6 [Source:NCBI gene (formerly Entrezgene);Acc:3283882]	2.52	1.333578	1.78E-09
NADH dehydrogenase subunit 5 [Source:NCBI gene (formerly Entrezgene);Acc:3283887]	3.134	1.648235	5.00E-09
NADH dehydrogenase subunit 6 [Source:NCBI gene (formerly Entrezgene);Acc:3283888]	2.567	1.36025	1.18E-08
NADH dehydrogenase subunit 4L [Source:NCBI gene (formerly Entrezgene);Acc:3283885]	3.194	1.675284	1.94E-08
Nuclear RNase P [Source:RFAM;Acc:RF00009]	0.137	-2.8693	2.28E-08
NADH dehydrogenase subunit 2 [Source:NCBI gene (formerly Entrezgene);Acc:3283878]	2.191	1.131426	2.56E-08
NADH dehydrogenase subunit 4 [Source:NCBI gene (formerly Entrezgene);Acc:3283886]	2.634	1.39736	1.16E-07
tubulin tyrosine ligase like 7 [Source:VGNC Symbol;Acc:VGNC:36495]	0.409	-1.28959	2.30E-06
complement C3 [Source:VGNC Symbol;Acc:VGNC:26638]	2.15	1.104469	2.38E-06
RNase MRP [Source:RFAM;Acc:RF00030]	0.148	-2.75783	6.62E-06
nucleolar protein 3 [Source:VGNC Symbol;Acc:VGNC:32156]	0.333	-1.58811	1.34E-05
DiGeorge syndrome critical region 6 like [Source:NCBI gene (formerly Entrezgene);Acc:100126445]	0.351	-1.51238	1.48E-05
homer scaffold protein 3 [Source:VGNC Symbol;Acc:VGNC:29903]	0.469	-1.0928	1.56E-05

fibronectin leucine rich transmembrane protein 2 [Source:VGNC Symbol;Acc:VGNC:29039]	3.648	1.867007	1.76E-05
NADH:ubiquinone oxidoreductase subunit A13 [Source:VGNC Symbol;Acc:VGNC:31946]	0.455	-1.13639	1.99E-05
aurora kinase A interacting protein 1 [Source:VGNC Symbol;Acc:VGNC:26350]	0.409	-1.28807	2.21E-05
mitochondrial ribosomal protein L41 [Source:VGNC Symbol;Acc:VGNC:31637]	0.377	-1.4056	2.52E-05
NADH:ubiquinone oxidoreductase core subunit S8 [Source:VGNC Symbol;Acc:VGNC:31973]	0.394	-1.34308	3.09E-05
endothelial differentiation related factor 1 [Source:VGNC Symbol;Acc:VGNC:28325]	0.434	-1.2028	3.97E-05
Metazoan signal recognition particle RNA [Source:RFAM;Acc:RF00017]	0.031	-5.0204	4.07E-05
ribosomal protein S15 [Source:VGNC Symbol;Acc:VGNC:34131]	0.418	-1.25849	4.14E-05
ras homolog family member D [Source:VGNC Symbol;Acc:VGNC:33947]	0.437	-1.19329	4.32E-05
ATP synthase membrane subunit e [Source:NCBI gene (formerly Entrezgene);Acc:338040]	0.466	-1.10281	4.48E-05
ECSIT signaling integrator [Source:VGNC Symbol;Acc:VGNC:28313]	0.416	-1.26664	4.53E-05
ribosomal protein L35 [Source:NCBI gene (formerly Entrezgene);Acc:515534]	0.443	-1.17356	4.73E-05
phosphohistidine phosphatase 1 [Source:NCBI gene (formerly Entrezgene);Acc:618691]	0.418	-1.25816	4.86E-05
MAPK regulated corepressor interacting protein 2 [Source:NCBI gene (formerly Entrezgene);Acc:618020]	0.379	-1.3984	5.15E-05

ribosomal protein L8 [Source:NCBI gene (formerly Entrezgene);Acc:535056]	0.403	-1.30958	5.16E-05
Rab acceptor 1 [Source:VGNC Symbol;Acc:VGNC:33666]	0.395	-1.34108	5.57E-05
Josephin domain containing 2 [Source:VGNC Symbol;Acc:VGNC:57352]	0.389	-1.36387	5.72E-05
ribosomal protein S28 [Source:VGNC Symbol;Acc:VGNC:34136]	0.437	-1.19385	6.01E-05
elongin B [Source:NCBI gene (formerly Entrezgene);Acc:617277]	0.392	-1.34984	6.07E-05
2'-deoxynucleoside 5'-phosphate N-hydrolase 1 [Source:VGNC Symbol;Acc:VGNC:28149]	0.371	-1.43053	6.09E-05
EMG1 N1-specific pseudouridine methyltransferase [Source:VGNC Symbol;Acc:VGNC:58013]	0.422	-1.24541	6.13E-05
H4 clustered histone 4 [Source:VGNC Symbol;Acc:VGNC:83562]	0.391	-1.35565	6.32E-05
transmembrane protein 160 [Source:VGNC Symbol;Acc:VGNC:35985]	0.364	-1.45768	6.59E-05
V-set and immunoglobulin domain containing 10 like [Source:VGNC Symbol;Acc:VGNC:36839]	0.441	-1.17989	6.64E-05
eukaryotic translation elongation factor 1 delta [Source:VGNC Symbol;Acc:VGNC:53861]	0.426	-1.2319	6.70E-05
MOB kinase activator 2 [Source:VGNC Symbol;Acc:VGNC:31543]	0.441	-1.18026	6.81E-05
ribosomal protein lateral stalk subunit P2 [Source:VGNC Symbol;Acc:VGNC:34119]	0.436	-1.19843	7.17E-05
mitochondrial ribosomal protein L40 [Source:VGNC Symbol;Acc:VGNC:31636]	0.385	-1.37714	7.19E-05
5', 3'-nucleotidase, cytosolic [Source:VGNC Symbol;Acc:VGNC:32289]	0.407	-1.29566	7.39E-05
COPI coat complex subunit epsilon	0.408	-1.2943	7.48E-05

[Source:VGNC Symbol;Acc:VGNC:27595]			
transmembrane protein 147 [Source:VGNC Symbol;Acc:VGNC:35978]	0.453	-1.14244	7.51E-05
coiled-coil-helix-coiled-coil-helix domain containing 6 [Source:VGNC Symbol;Acc:VGNC:27274]	0.409	-1.29152	7.82E-05
shisa family member 4 [Source:VGNC Symbol;Acc:VGNC:34600]	0.424	-1.23619	7.90E-05
NADH:ubiquinone oxidoreductase core subunit S7 [Source:VGNC Symbol;Acc:VGNC:31972]	0.433	-1.20669	8.23E-05
ribosomal protein L28 [Source:VGNC Symbol;Acc:VGNC:34114]	0.435	-1.19985	8.55E-05
ribosomal protein S5 [Source:VGNC Symbol;Acc:VGNC:34139]	0.426	-1.23149	8.57E-05
mitochondrial ribosomal protein L23 [Source:NCBI gene (formerly Entrezgene);Acc:508825]	0.429	-1.21947	8.66E-05
mitochondrial ribosomal protein L58 [Source:VGNC Symbol;Acc:VGNC:31652]	0.487	-1.03822	9.30E-05
zinc finger MYND-type containing 19 [Source:VGNC Symbol;Acc:VGNC:37205]	0.439	-1.18807	9.33E-05
deoxythymidylate kinase [Source:VGNC Symbol;Acc:VGNC:28240]	0.418	-1.25925	9.38E-05
multivesicular body subunit 12A [Source:VGNC Symbol;Acc:VGNC:31766]	0.445	-1.16856	9.45E-05
NADH:ubiquinone oxidoreductase subunit B7 [Source:VGNC Symbol;Acc:VGNC:31966]	0.433	-1.20712	9.55E-05
Metazoan signal recognition particle RNA [Source:RFAM;Acc:RF00017]	0.091	-3.46355	9.66E-05
uridine phosphorylase 1 [Source:VGNC	0.454	-1.13934	9.74E-05

Symbol;Acc:VGNC:36688]			
nudix hydrolase 14 [Source:VGNC Symbol;Acc:VGNC:32328]	0.369	-1.4381	9.96E-05
ribosomal protein S11 [Source:VGNC Symbol;Acc:VGNC:53041]	0.465	-1.1046	0.0001
trafficking protein particle complex 5 [Source:VGNC Symbol;Acc:VGNC:36291]	0.401	-1.31872	0.000101
peptidylprolyl cis/trans isomerase, NIMA-interacting 1 [Source:VGNC Symbol;Acc:VGNC:97301]	0.461	-1.11735	0.000102
presequence translocase associated motor 16 [Source:VGNC Symbol;Acc:VGNC:57355]	0.424	-1.23936	0.000105
PTOV1 extended AT-hook containing adaptor protein [Source:VGNC Symbol;Acc:VGNC:33522]	0.446	-1.1654	0.000111
CDGSH iron sulfur domain 3 [Source:VGNC Symbol;Acc:VGNC:27373]	0.408	-1.29198	0.000112
unconventional SNARE in the ER 1 [Source:NCBI gene (formerly Entrezgene);Acc:512890]	0.481	-1.05696	0.000114
LSM7 homolog, U6 small nuclear RNA and mRNA degradation associated [Source:VGNC Symbol;Acc:VGNC:31059]	0.5	-1.00079	0.000115
mitochondrial ribosomal protein L54 [Source:VGNC Symbol;Acc:VGNC:31649]	0.465	-1.10608	0.000116
ubiquitin conjugating enzyme E2 L6 [Source:VGNC Symbol;Acc:VGNC:36588]	0.462	-1.11394	0.000118
alkB homolog 7 [Source:VGNC Symbol;Acc:VGNC:25838]	0.447	-1.16028	0.000118
ribosomal protein L36 [Source:NCBI gene (formerly Entrezgene);Acc:768327]	0.464	-1.10747	0.000124
phosphomannomutase 1 [Source:VGNC Symbol;Acc:VGNC:33072]	0.48	-1.05762	0.000126

galactokinase 1 [Source:VGNC Symbol;Acc:VGNC:29219]	0.451	-1.14988	0.000128
mitochondrial contact site and cristae organizing system subunit 13 [Source:VGNC Symbol;Acc:VGNC:52730]	0.45	-1.15264	0.000129
mitochondrial ribosomal protein L17 [Source:NCBI gene (formerly Entrezgene);Acc:506988]	0.459	-1.12288	0.00013
RNA polymerase II, I and III subunit L [Source:VGNC Symbol;Acc:VGNC:33144]	0.498	-1.00678	0.00013
coiled-coil domain containing 85A [Source:VGNC Symbol;Acc:VGNC:26923]	0.487	-1.03797	0.000131
guided entry of tail-anchored proteins factor 3, ATPase [Source:VGNC Symbol;Acc:VGNC:96696]	0.429	-1.22057	0.000134
NADH:ubiquinone oxidoreductase core subunit V1 [Source:VGNC Symbol;Acc:VGNC:31974]	0.455	-1.13491	0.000136
nudix hydrolase 1 [Source:VGNC Symbol;Acc:VGNC:32325]	0.423	-1.24205	0.000139
ubiquitin conjugating enzyme E2 M [Source:VGNC Symbol;Acc:VGNC:36589]	0.473	-1.0808	0.000139
NADH:ubiquinone oxidoreductase complex assembly factor 8 [Source:HGNC Symbol;Acc:HGNC:33551]	0.46	-1.11907	0.000139
tubulin polymerization promoting protein family member 3 [Source:VGNC Symbol;Acc:VGNC:36260]	0.392	-1.35259	0.000141
basic leucine zipper ATF-like transcription factor 3 [Source:VGNC Symbol;Acc:VGNC:26427]	0.452	-1.14553	0.000141
LYR motif containing 4 [Source:VGNC Symbol;Acc:VGNC:31110]	0.458	-1.1262	0.000144
ERCC excision repair 1, endonuclease non-catalytic subunit	0.414	-1.2712	0.000145

[Source:VGNC Symbol;Acc:VGNC:28568]			
fission, mitochondrial 1 [Source:VGNC Symbol;Acc:VGNC:29014]	0.468	-1.09392	0.000146
RNA polymerase II, I and III subunit F [Source:VGNC Symbol;Acc:VGNC:33140]	0.471	-1.08496	0.000146
ATPase H+ transporting V1 subunit F [Source:NCBI gene (formerly Entrezgene);Acc:282405]	0.464	-1.10674	0.000149
mitochondrial ribosomal protein S2 [Source:VGNC Symbol;Acc:VGNC:31660]	0.457	-1.12949	0.00015
NADH:ubiquinone oxidoreductase subunit B11 [Source:NCBI gene (formerly Entrezgene);Acc:404161]	0.437	-1.19592	0.000152
BAF chromatin remodeling complex subunit BCL7C [Source:VGNC Symbol;Acc:VGNC:58362]	0.422	-1.24593	0.000153
mitochondrial ribosomal protein L2 [Source:VGNC Symbol;Acc:VGNC:31623]	0.447	-1.163	0.000153
protein phosphatase 1 regulatory inhibitor subunit 14A [Source:VGNC Symbol;Acc:VGNC:33226]	0.406	-1.30201	0.000155
HRas proto-onco, GTPase [Source:VGNC Symbol;Acc:VGNC:29951]	0.399	-1.32642	0.000157
exosome component 4 [Source:VGNC Symbol;Acc:VGNC:28659]	0.449	-1.15583	0.00016
cysteine rich DPF motif domain containing 1 [Source:VGNC Symbol;Acc:VGNC:27152]	0.457	-1.13102	0.00016
kielin cysteine rich BMP regulator [Source:VGNC Symbol;Acc:VGNC:53650]	0.334	-1.5842	0.000165
late endosomal/lysosomal adaptor, MAPK and MTOR activator 4 [Source:VGNC Symbol;Acc:VGNC:30782]	0.466	-1.10099	0.000167

PAXX non-homologous end joining factor [Source:VGNC Symbol;Acc:VGNC:32603]	0.395	-1.34038	0.000168
ribosomal protein S9 [Source:VGNC Symbol;Acc:VGNC:34152]	0.481	-1.05443	0.000169
mitochondrial ribosomal protein L12 [Source:NCBI gene (formerly Entrezgene);Acc:399560]	0.486	-1.04165	0.000171
TATA-box binding protein associated factor 10 [Source:VGNC Symbol;Acc:VGNC:35567]	0.459	-1.123	0.000174
mitochondrial ribosomal protein L28 [Source:VGNC Symbol;Acc:VGNC:31629]	0.44	-1.18413	0.000178
mitochondrial trans-2-enoyl-CoA reductase [Source:VGNC Symbol;Acc:VGNC:31345]	0.484	-1.04689	0.000181
coiled-coil domain containing 124 [Source:VGNC Symbol;Acc:VGNC:26840]	0.459	-1.12198	0.000182
MAPK regulated corepressor interacting protein 1 [Source:VGNC Symbol;Acc:VGNC:31322]	0.446	-1.16331	0.000182
nudix hydrolase 16 like 1 [Source:VGNC Symbol;Acc:VGNC:32331]	0.426	-1.23057	0.000182
RAS related [Source:VGNC Symbol;Acc:VGNC:49146]	0.476	-1.0721	0.000186
THO complex 6 [Source:VGNC Symbol;Acc:VGNC:35842]	0.476	-1.07019	0.000186
N-terminal Xaa-Pro-Lys N-methyltransferase 1 [Source:VGNC Symbol;Acc:VGNC:32303]	0.448	-1.15745	0.000197
anaphase promoting complex subunit 2 [Source:VGNC Symbol;Acc:VGNC:102815]	0.472	-1.08324	0.000197
mannosidase beta like [Source:VGNC Symbol;Acc:VGNC:31168]	0.497	-1.00934	0.000198
ribosomal protein L18a [Source:VGNC	0.471	-1.08605	0.0002

Symbol;Acc:VGNC:34112]			
parathymosin [Source:NCBI gene (formerly Entrezgene);Acc:613777]	0.443	-1.17602	0.000203
lysosomal trafficking regulator [Source:VGNC Symbol;Acc:VGNC:31115]	2.167	1.115856	0.000204
ribosomal protein L38 [Source:NCBI gene (formerly Entrezgene);Acc:615300]	0.496	-1.01238	0.000207
ribosomal protein L18 [Source:VGNC Symbol;Acc:VGNC:34111]	0.499	-1.00178	0.000208
YjeF N-terminal domain containing 3 [Source:VGNC Symbol;Acc:VGNC:97329]	0.386	-1.37279	0.000214
ADP ribosylation factor like GTPase 6 interacting protein 4 [Source:VGNC Symbol;Acc:VGNC:53889]	0.457	-1.13069	0.000214
ribonuclease/angiogenin inhibitor 1 [Source:NCBI gene (formerly Entrezgene);Acc:517087]	0.463	-1.11031	0.000216
polynucleotide kinase 3'-phosphatase [Source:VGNC Symbol;Acc:VGNC:33081]	0.499	-1.00291	0.000218
fibrillarin [Source:VGNC Symbol;Acc:VGNC:28879]	0.48	-1.05761	0.000221
ribosomal protein S20 [Source:VGNC Symbol;Acc:VGNC:34133]	0.464	-1.10634	0.000223
ferritin light chain [Source:NCBI gene (formerly Entrezgene);Acc:286861]	0.5	-1.00079	0.000224
ubiquinol-cytochrome c reductase core protein 1 [Source:VGNC Symbol;Acc:VGNC:36695]	0.473	-1.08093	0.000227
GADD45G interacting protein 1 [Source:VGNC Symbol;Acc:VGNC:29209]	0.439	-1.18655	0.000228
mono-ADP ribosylhydrolase 1 [Source:VGNC Symbol;Acc:VGNC:56272]	0.428	-1.22509	0.000235
reactive oxygen species modulator 1 [Source:VGNC	0.482	-1.05405	0.000235

Symbol;Acc:VGNC:34085]			
NME/NM23 nucleoside diphosphate kinase 3 [Source:VGNC Symbol;Acc:VGNC:32126]	0.366	-1.45157	0.000241
ATP synthase F1 subunit delta [Source:VGNC Symbol;Acc:VGNC:26302]	0.467	-1.09974	0.000244
D-dopachrome tautomerase [Source:NCBI gene (formerly Entrezgene);Acc:615999]	0.498	-1.00565	0.000245
peroxiredoxin like 2B [Source:VGNC Symbol;Acc:VGNC:28792]	0.44	-1.1851	0.000248
negative elongation factor complex member E [Source:VGNC Symbol;Acc:VGNC:32002]	0.498	-1.00684	0.000249
notchless homolog 1 [Source:VGNC Symbol;Acc:VGNC:32109]	0.462	-1.11306	0.000257
PNKD metallo-beta-lactamase domain containing [Source:NCBI gene (formerly Entrezgene);Acc:616561]	0.471	-1.08486	0.000257
HSPA (Hsp70) binding protein 1 [Source:NCBI gene (formerly Entrezgene);Acc:512757]	0.476	-1.07233	0.000264
mitochondrial ribosomal protein S25 [Source:VGNC Symbol;Acc:VGNC:31665]	0.448	-1.1585	0.000273
WW domain binding protein 1 [Source:VGNC Symbol;Acc:VGNC:36870]	0.491	-1.0269	0.000276
histidine triad nucleotide binding protein 2 [Source:VGNC Symbol;Acc:VGNC:29855]	0.473	-1.0788	0.00028
tubulin alpha 3d [Source:HGNC Symbol;Acc:HGNC:24071]	0.44	-1.1846	0.000281
coronin 1B [Source:VGNC Symbol;Acc:VGNC:27621]	0.474	-1.07724	0.000283
ribosomal protein L7a [Source:VGNC Symbol;Acc:VGNC:49957]	0.467	-1.09888	0.000283
proteasome 20S subunit beta 6	0.471	-1.08701	0.000285

[Source:VGNC Symbol;Acc:VGNC:33450]			
tRNA methyltransferase 2 homolog A [Source:VGNC Symbol;Acc:VGNC:36372]	0.496	-1.01104	0.000289
catechol-O-methyltransferase domain containing 1 [Source:VGNC Symbol;Acc:VGNC:27591]	0.468	-1.09648	0.000293
dihydrouridine synthase 3 like [Source:VGNC Symbol;Acc:VGNC:103057]	0.475	-1.07276	0.000294
zinc finger protein 593 [Source:VGNC Symbol;Acc:VGNC:56158]	0.486	-1.04169	0.000295
signal sequence receptor subunit 4 [Source:VGNC Symbol;Acc:VGNC:35322]	0.491	-1.02604	0.000295
NOP53 ribosome biosis factor [Source:VGNC Symbol;Acc:VGNC:32168]	0.469	-1.09362	0.000296
guanylate kinase 1 [Source:VGNC Symbol;Acc:VGNC:29725]	0.491	-1.02652	0.000297
zinc finger HIT-type containing 2 [Source:VGNC Symbol;Acc:VGNC:37359]	0.479	-1.061	0.000303
eukaryotic translation initiation factor 3 subunit K [Source:VGNC Symbol;Acc:VGNC:28401]	0.493	-1.02124	0.000304
cysteine rich protein 1 [Source:NCBI gene (formerly Entrezgene);Acc:574093]	0.499	-1.00181	0.000305
Metazoan signal recognition particle RNA [Source:RFAM;Acc:RF00017]	0.058	-4.1041	0.000305
Fanconi anemia core complex associated protein 20 [Source:NCBI gene (formerly Entrezgene);Acc:508039]	0.487	-1.03819	0.000307
host cell factor C1 regulator 1 [Source:VGNC Symbol;Acc:VGNC:29772]	0.477	-1.06652	0.000308
MISP family member 3 [Source:VGNC	0.436	-1.19748	0.000315

Symbol;Acc:VGNC:55217]			
mitochondrial nucleoid factor 1 [Source:NCBI gene (formerly Entrezgene);Acc:100607974]	0.465	-1.1038	0.000315
cytochrome c oxidase subunit 4I1 [Source:VGNC Symbol;Acc:VGNC:27634]	0.472	-1.08407	0.000315
vesicle associated membrane protein 5 [Source:VGNC Symbol;Acc:VGNC:36759]	0.452	-1.14482	0.000318
GDP-L-fucose synthase [Source:VGNC Symbol;Acc:VGNC:36450]	0.474	-1.0764	0.000319
arginine vasopressin induced 1 [Source:VGNC Symbol;Acc:VGNC:26357]	0.479	-1.06243	0.000319
mitogen-activated protein kinase 8 interacting protein 1 [Source:VGNC Symbol;Acc:VGNC:31224]	0.473	-1.08113	0.00032
ubiquitin A-52 residue ribosomal protein fusion product 1 [Source:VGNC Symbol;Acc:VGNC:49988]	0.484	-1.04547	0.000322
NADH:ubiquinone oxidoreductase subunit A11 [Source:NCBI gene (formerly Entrezgene);Acc:326346]	0.448	-1.15767	0.000323
eukaryotic translation elongation factor 1 gamma [Source:VGNC Symbol;Acc:VGNC:28334]	0.478	-1.06627	0.000325
family with sequence similarity 8 member A1 [Source:VGNC Symbol;Acc:VGNC:28844]	2.011	1.00757	0.000326
methyltransferase like 26 [Source:VGNC Symbol;Acc:VGNC:31410]	0.463	-1.11171	0.000329
ADP ribosylation factor like GTPase 2 [Source:VGNC Symbol;Acc:VGNC:97240]	0.467	-1.09817	0.000335
tRNA methyltransferase 61A [Source:VGNC Symbol;Acc:VGNC:36375]	0.457	-1.12985	0.000341
fucose mutarotase [Source:VGNC Symbol;Acc:VGNC:29145]	0.461	-1.11688	0.000343

SS nuclear autoantigen 1 [Source:VGNC Symbol;Acc:VGNC:35316]	0.494	-1.01765	0.000346
isocitrate dehydrogenase (NAD(+)) 3 non-catalytic subunit gamma [Source:VGNC Symbol;Acc:VGNC:54635]	0.48	-1.0592	0.000347
FAU ubiquitin like and ribosomal protein S30 fusion [Source:NCBI gene (formerly Entrezgene);Acc:282847]	0.5	-1.00105	0.000351
macrophage migration inhibitory factor [Source:VGNC Symbol;Acc:VGNC:49557]	0.481	-1.05561	0.000352
N-methylpurine DNA glycosylase [Source:VGNC Symbol;Acc:VGNC:58391]	0.483	-1.05104	0.000355
mitotic arrest deficient 2 like 2 [Source:VGNC Symbol;Acc:VGNC:31132]	0.487	-1.03892	0.000356
proteasome assembly chaperone 4 [Source:VGNC Symbol;Acc:VGNC:33481]	0.377	-1.40545	0.000357
ribonuclease H2 subunit A [Source:VGNC Symbol;Acc:VGNC:34000]	0.48	-1.05901	0.000363
Peroxisome Proliferator-activated Receptor gamma	43.094	5.429429	0.000366
NmrA like redox sensor 1 [Source:VGNC Symbol;Acc:VGNC:32134]	0.483	-1.04945	0.000372
nth like DNA glycosylase 1 [Source:VGNC Symbol;Acc:VGNC:32301]	0.463	-1.10978	0.000375
hydroxysteroid 17-beta dehydrogenase 10 [Source:VGNC Symbol;Acc:VGNC:29969]	0.468	-1.09691	0.000376
argininosuccinate lyase [Source:NCBI gene (formerly Entrezgene);Acc:512771]	0.494	-1.01615	0.000376
syntrophin alpha 1 [Source:VGNC Symbol;Acc:VGNC:35083]	0.476	-1.07119	0.000384
Ras association domain family member 7 [Source:VGNC	0.481	-1.05481	0.000386

Symbol;Acc:VGNC:33763]			
MTOR associated protein, LST8 homolog [Source:VGNC Symbol;Acc:VGNC:31507]	0.495	-1.01466	0.000389
eukaryotic translation initiation factor 6 [Source:VGNC Symbol;Acc:VGNC:57006]	0.475	-1.07368	0.000392
dual specificity phosphatase 15 [Source:HGNC Symbol;Acc:HGNC:16236]	0.47	-1.09016	0.000396
Yip1 interacting factor homolog B, membrane trafficking protein [Source:VGNC Symbol;Acc:VGNC:37024]	0.474	-1.07845	0.00041
FKBP prolyl isomerase 8 [Source:NCBI gene (formerly Entrezgene);Acc:512356]	0.471	-1.08624	0.000417
zinc finger protein 771 [Source:VGNC Symbol;Acc:VGNC:37342]	0.478	-1.06504	0.000426
zinc finger protein 575 [Source:NCBI gene (formerly Entrezgene);Acc:504571]	0.428	-1.22439	0.000429
tumor protein p63 regulated 1 like [Source:VGNC Symbol;Acc:VGNC:36262]	0.478	-1.06347	0.00043
ribosomal protein S6 kinase B2 [Source:VGNC Symbol;Acc:VGNC:34147]	0.498	-1.00722	0.000431
dolichyl-phosphate mannosyltransferase subunit 3, regulatory [Source:VGNC Symbol;Acc:VGNC:28185]	0.454	-1.13837	0.000432
cyclin dependent kinase 3 [Source:VGNC Symbol;Acc:VGNC:55794]	0.48	-1.0595	0.000434
mitochondrial ribosomal protein S26 [Source:VGNC Symbol;Acc:VGNC:31666]	0.5	-1.00007	0.000434
NADH:ubiquinone oxidoreductase subunit A3 [Source:NCBI gene (formerly Entrezgene);Acc:338064]	0.489	-1.03292	0.000437
6-phosphogluconolactonase [Source:VGNC	0.468	-1.09668	0.000442

Symbol;Acc:VGNC:32790]			
5'-nucleotidase domain containing 2 [Source:VGNC Symbol;Acc:VGNC:32295]	0.482	-1.05438	0.000443
adenine phosphoribosyltransferase [Source:VGNC Symbol;Acc:VGNC:26042]	0.467	-1.09975	0.000445
G protein subunit beta 1 like [Source:VGNC Symbol;Acc:VGNC:29458]	0.48	-1.05896	0.000446
mitochondrial ribosomal protein L24 [Source:VGNC Symbol;Acc:VGNC:31627]	0.485	-1.0436	0.000453
protease associated domain containing 1 [Source:VGNC Symbol;Acc:VGNC:33282]	0.482	-1.05206	0.000456
protein tyrosine phosphatase receptor type C associated protein [Source:VGNC Symbol;Acc:VGNC:33547]	0.328	-1.60825	0.000457
crystallin beta B1 [Source:VGNC Symbol;Acc:VGNC:27736]	0.417	-1.26349	0.000457
chromosome 25 C7orf50 homolog [Source:NCBI gene (formerly Entrezgene);Acc:522840]	0.496	-1.01078	0.000466
Src homology 2 domain containing F [Source:VGNC Symbol;Acc:VGNC:34596]	0.487	-1.03787	0.000498
thioredoxin reductase 2 [Source:VGNC Symbol;Acc:VGNC:36546]	0.497	-1.00904	0.000508
deoxyhypusine hydroxylase [Source:VGNC Symbol;Acc:VGNC:28164]	0.49	-1.02944	0.000518
2-oxoglutarate and iron dependent oxygenase domain containing 2 [Source:VGNC Symbol;Acc:VGNC:32409]	0.462	-1.11418	0.000531
pseudouridine synthase like 1 [Source:VGNC Symbol;Acc:VGNC:33578]	0.496	-1.01023	0.000547
TCF3 fusion partner [Source:VGNC Symbol;Acc:VGNC:35795]	0.46	-1.12025	0.000561

CUE domain containing 2 [Source:VGNC Symbol;Acc:VGNC:55945]	0.49	-1.02935	0.000565
protein tyrosine phosphatase mitochondrial 1 [Source:VGNC Symbol;Acc:VGNC:33528]	0.476	-1.07033	0.000568
SLC2A4 regulator [Source:VGNC Symbol;Acc:VGNC:57026]	0.486	-1.0407	0.000571
acyl-CoA thioesterase 8 [Source:VGNC Symbol;Acc:VGNC:25550]	0.491	-1.02494	0.000573
tubulin polyglutamylase complex subunit 1 [Source:VGNC Symbol;Acc:VGNC:36247]	0.473	-1.0815	0.000578
dynein 2 intermediate chain 2 [Source:VGNC Symbol;Acc:VGNC:36893]	0.493	-1.02152	0.000581
THAP domain containing 3 [Source:VGNC Symbol;Acc:VGNC:35822]	0.457	-1.12858	0.000589
acetyl-CoA acyltransferase 1 [Source:VGNC Symbol;Acc:VGNC:25517]	0.495	-1.01499	0.000605
UBX domain protein 6 [Source:VGNC Symbol;Acc:VGNC:36628]	0.496	-1.01105	0.000605
ADP ribosylation factor GTPase activating protein 1 [Source:VGNC Symbol;Acc:VGNC:56176]	0.489	-1.03297	0.000606
H2B clustered histone 6 [Source:VGNC Symbol;Acc:VGNC:83564]	0.066	-3.93187	0.000611
O-6-methylguanine-DNA methyltransferase [Source:HGNC Symbol;Acc:HGNC:7059]	0.487	-1.03772	0.00062
B9 domain containing 2 [Source:VGNC Symbol;Acc:VGNC:26397]	0.478	-1.06595	0.000621
5.8S ribosomal RNA [Source:RFAM;Acc:RF00002]	0.075	-3.73112	0.000627
cysteinyl-tRNA synthetase 2, mitochondrial [Source:VGNC Symbol;Acc:VGNC:49547]	0.486	-1.0413	0.000638
stress associated endoplasmic	0.464	-1.10697	0.000638

reticulum protein family member 2 [Source:NCBI gene (formerly Entrezgene);Acc:613627]			
StAR related lipid transfer domain containing 10 [Source:VGNC Symbol;Acc:VGNC:35361]	0.488	-1.03547	0.000648
ArfGAP with RhoGAP domain, ankyrin repeat and PH domain 2 [Source:VGNC Symbol;Acc:VGNC:26055]	3.048	1.60771	0.00065
RNA polymerase II subunit I [Source:VGNC Symbol;Acc:VGNC:33143]	0.466	-1.10311	0.000653
thymocyte nuclear protein 1 [Source:VGNC Symbol;Acc:VGNC:35857]	0.498	-1.00684	0.000668
trafficking protein particle complex 6A [Source:VGNC Symbol;Acc:VGNC:36292]	0.432	-1.21188	0.000669
required for excision 1-B domain containing [Source:VGNC Symbol;Acc:VGNC:52875]	0.498	-1.0054	0.000671
ubiquinol-cytochrome c reductase, complex III subunit XI [Source:NCBI gene (formerly Entrezgene);Acc:281570]	0.496	-1.01214	0.000677
mitotic spindle organizing protein 2B [Source:NCBI gene (formerly Entrezgene);Acc:787548]	0.457	-1.12932	0.0007
BCS1 homolog, ubiquinol-cytochrome c reductase complex chaperone [Source:VGNC Symbol;Acc:VGNC:26456]	0.493	-1.01997	0.000701
mitochondrial ribosomal protein L57 [Source:VGNC Symbol;Acc:VGNC:31651]	0.49	-1.02788	0.000708
mitochondrial ribosomal protein S34 [Source:VGNC Symbol;Acc:VGNC:31672]	0.473	-1.07917	0.000716
mitogen-activated protein kinase 12 [Source:VGNC Symbol;Acc:VGNC:31215]	0.485	-1.04377	0.000718
DNA polymerase delta 4, accessory subunit [Source:NCBI gene	0.5	-1.00129	0.000721

(formerly Entrezgene);Acc:617899]			
MAX dimerization protein 3 [Source:VGNC Symbol;Acc:VGNC:55224]	0.47	-1.08941	0.00075
methylthioribose-1-phosphate isomerase 1 [Source:VGNC Symbol;Acc:VGNC:31604]	0.497	-1.0091	0.000764
tubulin, alpha 3e [Source:NCBI gene (formerly Entrezgene);Acc:534900]	0.489	-1.03164	0.000778
VPS51 subunit of GARP complex [Source:VGNC Symbol;Acc:VGNC:36827]	0.497	-1.00952	0.000821
aryl hydrocarbon receptor interacting protein [Source:VGNC Symbol;Acc:VGNC:25767]	0.49	-1.02863	0.000823
transducin beta like 3 [Source:VGNC Symbol;Acc:VGNC:35659]	0.483	-1.05108	0.00085
adhesion G protein-coupled receptor V1 [Source:VGNC Symbol;Acc:VGNC:25673]	2.198	1.136398	0.000854
ubiquitin like 7 [Source:VGNC Symbol;Acc:VGNC:50128]	0.497	-1.00767	0.000864
nuclear protein 2, transcriptional regulator [Source:NCBI gene (formerly Entrezgene);Acc:614047]	0.47	-1.09004	0.00089
RAN guanine nucleotide release factor [Source:VGNC Symbol;Acc:VGNC:33714]	0.444	-1.17092	0.000901
proline rich 5 [Source:VGNC Symbol;Acc:VGNC:33400]	0.468	-1.09472	0.000925
ASPSCR1 tether for SLC2A4, UBX domain containing [Source:VGNC Symbol;Acc:VGNC:97242]	0.487	-1.03796	0.000942
S100 calcium binding protein A5 [Source:VGNC Symbol;Acc:VGNC:34245]	0.456	-1.13444	0.000964
7SK RNA [Source:RFAM;Acc:RF00100]	0.181	-2.46875	0.000976
BOP1 ribosomal biosis factor [Source:VGNC Symbol;Acc:VGNC:26540]	0.49	-1.02987	0.001032

Vertebrate telomerase RNA [Source:RFAM;Acc:RF00024]	0.154	-2.70077	0.001033
coenzyme Q4 [Source:VGNC Symbol;Acc:VGNC:59211]	0.498	-1.00589	0.001045
tumor suppressing subtransferable candidate 4 [Source:NCBI gene (formerly Entrezgene);Acc:509559]	0.498	-1.00458	0.001085
cystatin E/M [Source:NCBI gene (formerly Entrezgene);Acc:503685]	0.485	-1.04374	0.001101
HCLS1 associated protein X-1 [Source:VGNC Symbol;Acc:VGNC:29764]	0.477	-1.06655	0.001114
aminoacylase 1 [Source:NCBI gene (formerly Entrezgene);Acc:768058]	0.498	-1.00512	0.001115
family with sequence similarity 131 member C [Source:VGNC Symbol;Acc:VGNC:28729]	0.469	-1.09083	0.001137
hydroxysteroid 17-beta dehydrogenase 8 [Source:VGNC Symbol;Acc:VGNC:29977]	0.465	-1.10379	0.00115
dual specificity phosphatase 23 [Source:VGNC Symbol;Acc:VGNC:28257]	0.411	-1.28194	0.001175
TEN1 subunit of CST complex [Source:VGNC Symbol;Acc:VGNC:54498]	0.448	-1.15806	0.001187
H4 clustered histone 3 [Source:VGNC Symbol;Acc:VGNC:84553]	0.023	-5.46124	0.001199
guanidinoacetate N-methyltransferase [Source:VGNC Symbol;Acc:VGNC:29243]	0.486	-1.04056	0.00121
H3 clustered histone 13 [Source:VGNC Symbol;Acc:VGNC:83595]	0.194	-2.36796	0.001213
selenoprotein O [Source:VGNC Symbol;Acc:VGNC:58445]	0.486	-1.0419	0.001252
PDZ and LIM domain 4 [Source:VGNC Symbol;Acc:VGNC:32710]	0.218	-2.20069	0.001257
nudix hydrolase 18 [Source:VGNC Symbol;Acc:VGNC:32333]	0.477	-1.06777	0.001272
angiopoietin like 6 [Source:VGNC	0.42	-1.25133	0.001279

Symbol;Acc:VGNC:25894]			
dynein axonemal heavy chain 7 [Source:VGNC Symbol;Acc:VGNC:50052]	3.45	1.786735	0.001285
troponin T2, cardiac type [Source:VGNC Symbol;Acc:VGNC:36195]	0.494	-1.01749	0.00136
family with sequence similarity 214 member A [Source:VGNC Symbol;Acc:VGNC:28793]	2.356	1.236463	0.001389
transcription elongation factor A2 [Source:VGNC Symbol;Acc:VGNC:35680]	0.448	-1.15689	0.00139
F-box protein 2 [Source:VGNC Symbol;Acc:VGNC:28897]	0.475	-1.07438	0.001425
H2B clustered histone 13 [Source:VGNC Symbol;Acc:VGNC:83581]	0.287	-1.80029	0.001607
jumonji domain containing 7 [Source:VGNC Symbol;Acc:VGNC:55118]	0.453	-1.14141	0.001729
multiple EGF like domains 9 [Source:VGNC Symbol;Acc:VGNC:31377]	2.06	1.042717	0.001745
proline rich 16 [Source:HGNC Symbol;Acc:HGNC:29654]	0.353	-1.50187	0.001769
zinc finger protein 112 [Source:VGNC Symbol;Acc:VGNC:52980]	2.084	1.059318	0.001823
H2B clustered histone 18 [Source:VGNC Symbol;Acc:VGNC:83596]	0.13	-2.94705	0.00186
serine/threonine kinase 32C [Source:VGNC Symbol;Acc:VGNC:35395]	0.489	-1.03142	0.001998
copper chaperone for superoxide dismutase [Source:VGNC Symbol;Acc:VGNC:26991]	0.499	-1.00321	0.002084
upstream transcription factor family member 3 [Source:VGNC Symbol;Acc:VGNC:53612]	2.102	1.071665	0.00223
Metazoan signal recognition particle RNA [Source:RFAM;Acc:RF00017]	0.033	-4.9407	0.002253

Metazoan signal recognition particle RNA [Source:RFAM;Acc:RF00017]	0.136	-2.88328	0.002288
poly(rC) binding protein 3 [Source:VGNC Symbol;Acc:VGNC:53597]	0.462	-1.11457	0.002291
caveolae associated protein 2 [Source:VGNC Symbol;Acc:VGNC:26803]	2.545	1.347556	0.002396
complement C8 gamma chain [Source:NCBI gene (formerly Entrezgene);Acc:517356]	0.413	-1.27542	0.002485
secernin 2 [Source:VGNC Symbol;Acc:VGNC:34367]	0.495	-1.0153	0.002597
ER membrane protein complex subunit 9 [Source:VGNC Symbol;Acc:VGNC:28468]	0.478	-1.06363	0.002676
LSM10, U7 small nuclear RNA associated [Source:VGNC Symbol;Acc:VGNC:31051]	0.493	-1.0217	0.002758
KIAA2026 [Source:VGNC Symbol;Acc:VGNC:59197]	2.05	1.035446	0.002922
H2B clustered histone 14 [Source:VGNC Symbol;Acc:VGNC:83583]	0.239	-2.06593	0.003288
inka box actin regulator 1 [Source:VGNC Symbol;Acc:VGNC:28790]	0.036	-4.78982	0.00348
Kruppel like factor 9 [Source:VGNC Symbol;Acc:VGNC:30632]	2.064	1.045588	0.003646
H2A clustered histone 20 [Source:VGNC Symbol;Acc:VGNC:83605]	0.346	-1.52969	0.003648
tet methylcytosine dioxygenase 2 [Source:VGNC Symbol;Acc:VGNC:35757]	2.023	1.016162	0.004268
H2A clustered histone 18 [Source:VGNC Symbol;Acc:VGNC:83599]	0.49	-1.0278	0.004275
Rac family small GTPase 2 [Source:VGNC Symbol;Acc:VGNC:49061]	0.045	-4.47471	0.004322
adenine nucleotide translocase	0.48	-1.06033	0.004505

lysine methyltransferase [Source:NCBI gene (formerly Entrezgene);Acc:530342]			
apolipoprotein M [Source:VGNC Symbol;Acc:VGNC:26034]	0.485	-1.0433	0.004595
WAP four-disulfide core domain 3 [Source:VGNC Symbol;Acc:VGNC:57034]	0.493	-1.02072	0.004621
zinc finger and BTB domain containing 34 [Source:VGNC Symbol;Acc:VGNC:37074]	2.8	1.485576	0.004778
neuroligin 1 [Source:VGNC Symbol;Acc:VGNC:58395]	2.065	1.046346	0.004938
H2A clustered histone 6 [Source:VGNC Symbol;Acc:VGNC:83561]	0.352	-1.50703	0.005392
Small nucleolar RNA U3 [Source:RFAM;Acc:RF00012]	0.173	-2.52802	0.005479
Small nucleolar RNA U3 [Source:RFAM;Acc:RF00012]	0.173	-2.52802	0.005479
glypican 6 [Source:NCBI gene (formerly Entrezgene);Acc:536153]	0.223	-2.16203	0.005608
pleckstrin homology like domain family A member 2 [Source:VGNC Symbol;Acc:VGNC:32836]	0.419	-1.25389	0.00565
selenocysteine lyase [Source:VGNC Symbol;Acc:VGNC:55677]	0.496	-1.01256	0.00578
acid phosphatase 4 [Source:VGNC Symbol;Acc:VGNC:52175]	0.357	-1.48721	0.005785
rhophilin Rho GTPase binding protein 1 [Source:VGNC Symbol;Acc:VGNC:33956]	0.465	-1.10318	0.005833
midkine [Source:VGNC Symbol;Acc:VGNC:49556]	7.087	2.825125	0.006561
chromosome 1 open reading frame 53 [Source:HGNC Symbol;Acc:HGNC:30003]	0.45	-1.15155	0.006675
troponin T1, slow skeletal type [Source:VGNC Symbol;Acc:VGNC:36194]	0.27	-1.89118	0.00702
threonine synthase like 1 [Source:VGNC Symbol;Acc:VGNC:35838]	2.155	1.107597	0.007222

REST corepressor 2 [Source:VGNC Symbol;Acc:VGNC:33835]	0.489	-1.03135	0.00733
zinc finger and BTB domain containing 45 [Source:HGNC Symbol;Acc:HGNC:23715]	0.467	-1.09971	0.007534
granzyme M [Source:VGNC Symbol;Acc:VGNC:29734]	0.218	-2.19501	0.007552
lymphocyte antigen 75 [Source:NCBI gene (formerly Entrezgene);Acc:407236]	2.159	1.110149	0.007724
C-type lectin domain containing 11A [Source:VGNC Symbol;Acc:VGNC:27422]	0.455	-1.13637	0.007726
serine/threonine/tyrosine interacting like 1 [Source:VGNC Symbol;Acc:VGNC:35453]	0.439	-1.18794	0.00839
transcriptional repressor GATA binding 1 [Source:VGNC Symbol;Acc:VGNC:36395]	2.055	1.039203	0.0084
glutamate ionotropic receptor delta type subunit 2 [Source:HGNC Symbol;Acc:HGNC:4576]	2.118	1.082867	0.008707
zinc finger protein 614 [Source:VGNC Symbol;Acc:VGNC:37313]	2.103	1.072554	0.009733
neurexophilin 4 [Source:VGNC Symbol;Acc:VGNC:32383]	0.402	-1.31333	0.010535
calcium responsive transcription factor [Source:VGNC Symbol;Acc:VGNC:26765]	2.13	1.090537	0.010819
actinin alpha 3 [Source:VGNC Symbol;Acc:VGNC:53654]	0.323	-1.62927	0.011038
U1 spliceosomal RNA [Source:RFAM;Acc:RF00003]	0.106	-3.24461	0.011436
platelet derived growth factor D [Source:VGNC Symbol;Acc:VGNC:32691]	2.095	1.067141	0.011773
double C2 domain alpha [Source:VGNC Symbol;Acc:VGNC:28153]	0.057	-4.12893	0.011902
cyclin G2 [Source:VGNC Symbol;Acc:VGNC:26970]	2.282	1.190351	0.011907
CUB and Sushi multiple domains 3	0.387	-1.36955	0.012052

[Source:HGNC Symbol;Acc:HGNC:19291]			
Rho GTPase activating protein 42 [Source:VGNC Symbol;Acc:VGNC:26096]	2.003	1.002457	0.012526
H2A clustered histone 10 [Source:VGNC Symbol;Acc:VGNC:83576]	0.052	-4.25981	0.012683
HGF activator [Source:VGNC Symbol;Acc:VGNC:53764]	0.461	-1.1184	0.013131
H2A clustered histone 14 [Source:VGNC Symbol;Acc:VGNC:83584]	0.436	-1.19793	0.013141
cysteine rich tail 1 [Source:VGNC Symbol;Acc:VGNC:50272]	0.385	-1.37703	0.013604
BTB domain containing 8 [Source:HGNC Symbol;Acc:HGNC:21019]	2.312	1.209332	0.01389
coiled-coil domain containing 186 [Source:VGNC Symbol;Acc:VGNC:26881]	2.143	1.099797	0.01398
proline rich 22 [Source:VGNC Symbol;Acc:VGNC:52867]	0.221	-2.17982	0.01403
H1.2 linker histone, cluster member [Source:VGNC Symbol;Acc:VGNC:83558]	0.402	-1.31355	0.014036
RELT like 2 [Source:VGNC Symbol;Acc:VGNC:33861]	0.44	-1.18543	0.014036
mesoderm posterior bHLH transcription factor 1 [Source:HGNC Symbol;Acc:HGNC:29658]	0.484	-1.0463	0.014964
YOD1 deubiquitinase [Source:VGNC Symbol;Acc:VGNC:37033]	2.263	1.178426	0.015556
H2A clustered histone 11 [Source:VGNC Symbol;Acc:VGNC:83577]	0.031	-4.99713	0.015994
chondroitin sulfate synthase 3 [Source:VGNC Symbol;Acc:VGNC:27351]	2.391	1.257574	0.016537
Small nucleolar RNA U3 [Source:RFAM;Acc:RF00012]	0.183	-2.45057	0.016663
phosphodiesterase 4D	2.016	1.011733	0.01673

[Source:VGNC Symbol;Acc:VGNC:32677]			
fibrinogen silencer binding protein [Source:VGNC Symbol;Acc:VGNC:55754]	19.648	4.296311	0.01715
transmembrane protein 37 [Source:VGNC Symbol;Acc:VGNC:36079]	0.498	-1.00573	0.018207
tubulin alpha 8 [Source:VGNC Symbol;Acc:VGNC:36504]	0.325	-1.61975	0.01828
ribonucleoprotein, PTB binding 2 [Source:VGNC Symbol;Acc:VGNC:33766]	2.157	1.109246	0.01831
ring finger protein 208 [Source:VGNC Symbol;Acc:VGNC:34049]	0.459	-1.1231	0.018366
proline rich 7, synaptic [Source:VGNC Symbol;Acc:VGNC:33402]	0.381	-1.3924	0.01927
H2B clustered histone 12 [Source:VGNC Symbol;Acc:VGNC:83582]	0.457	-1.12865	0.019568
fyn related Src family tyrosine kinase [Source:VGNC Symbol;Acc:VGNC:29112]	2.061	1.043036	0.019632
TNF superfamily member 10 [Source:VGNC Symbol;Acc:VGNC:36172]	3.842	1.941753	0.020366
meiotic double-stranded break formation protein 1 [Source:VGNC Symbol;Acc:VGNC:31378]	0.397	-1.33345	0.020537
SH2 domain containing 7 [Source:VGNC Symbol;Acc:VGNC:34562]	9.365	3.227313	0.020843
MyoD family inhibitor [Source:VGNC Symbol;Acc:VGNC:31329]	0.474	-1.07555	0.021399
family with sequence similarity 229 member B [Source:VGNC Symbol;Acc:VGNC:28804]	0.441	-1.18054	0.021609
solute carrier family 2 member 9 [Source:VGNC Symbol;Acc:VGNC:34804]	2.106	1.074334	0.021895
U4 spliceosomal RNA	0.193	-2.37225	0.021946

[Source:RFAM;Acc:RF00015]			
H2A clustered histone 17 [Source:VGNC Symbol;Acc:VGNC:83586]	0.076	-3.72674	0.022532
leucine rich repeat neuronal 3 [Source:VGNC Symbol;Acc:VGNC:31040]	2.703	1.434606	0.024612
TSSK6 activating cochaperone [Source:NCBI gene (formerly Entrezgene);Acc:767912]	0.468	-1.09413	0.024962
coagulation factor XI [Source:VGNC Symbol;Acc:VGNC:28678]	2.254	1.172764	0.025222
synaptonemal complex protein 2 [Source:VGNC Symbol;Acc:VGNC:35509]	3.348	1.743336	0.025395
inositol monophosphatase 2 [Source:VGNC Symbol;Acc:VGNC:30185]	0.419	-1.25475	0.026592
mitogen-activated protein kinase kinase kinase 13 [Source:VGNC Symbol;Acc:VGNC:31191]	2.028	1.020319	0.026807
N-acetyltransferase 14 (putative) [Source:NCBI gene (formerly Entrezgene);Acc:532809]	0.498	-1.00505	0.026964
ubiquitin specific peptidase 49 [Source:VGNC Symbol;Acc:VGNC:36734]	3.501	1.807758	0.027875
H3 clustered histone 6 [Source:VGNC Symbol;Acc:VGNC:84555]	0.183	-2.45325	0.028242
spondin 2 [Source:VGNC Symbol;Acc:VGNC:35228]	0.481	-1.05525	0.028273
hook microtubule tethering protein 1 [Source:VGNC Symbol;Acc:VGNC:29905]	2.006	1.004639	0.029021
aldo-keto reductase family 1 member E2 [Source:VGNC Symbol;Acc:VGNC:57038]	0.281	-1.83093	0.029625
myosin light chain, phosphorylatable, fast skeletal muscle [Source:VGNC Symbol;Acc:VGNC:31809]	0.072	-3.78713	0.030024
sperm flagellar 1 [Source:VGNC	0.493	-1.01919	0.030353

Symbol;Acc:VGNC:35201]			
metaxin 3 [Source:VGNC Symbol;Acc:VGNC:55125]	2.3	1.201364	0.030437
syncytin-Rum1 [Source:NCBI gene (formerly Entrezgene);Acc:104973909]	2.675	1.419321	0.030794
coiled-coil glutamate rich protein 2 [Source:VGNC Symbol;Acc:VGNC:52185]	0.285	-1.80902	0.030844
H2A clustered histone 8 [Source:VGNC Symbol;Acc:VGNC:83570]	0.095	-3.39938	0.03217
abhydrolase domain containing 1 [Source:VGNC Symbol;Acc:VGNC:25487]	0.431	-1.21526	0.032391
complement C1q like 1 [Source:VGNC Symbol;Acc:VGNC:26620]	0.496	-1.0125	0.032727
lysophosphatidic acid receptor 5 [Source:VGNC Symbol;Acc:VGNC:30959]	0.069	-3.85199	0.033108
C-C motif chemokine ligand 25 [Source:VGNC Symbol;Acc:VGNC:26951]	0.463	-1.10999	0.033229
laminin subunit alpha 1 [Source:VGNC Symbol;Acc:VGNC:30769]	2.295	1.198194	0.033513
solute carrier family 34 member 3 [Source:VGNC Symbol;Acc:VGNC:58411]	0.406	-1.3002	0.034416
TNFAIP3 interacting protein 3 [Source:VGNC Symbol;Acc:VGNC:36181]	0.385	-1.37537	0.035047
hyperpolarization activated cyclic nucleotide gated potassium channel 4 [Source:VGNC Symbol;Acc:VGNC:29779]	0.348	-1.52415	0.035802
U1 spliceosomal RNA [Source:RFAM;Acc:RF00003]	0.03	-5.07496	0.036135
cyclin dependent kinase like 5 [Source:VGNC Symbol;Acc:VGNC:56922]	11.423	3.513919	0.036349
H2A clustered histone 12 [Source:VGNC	0.287	-1.80117	0.036802

Symbol;Acc:VGNC:83580]			
zonadhesin [Source:VGNC Symbol;Acc:VGNC:99806]	0.108	-3.20748	0.0375
zinc finger protein 529 [Source:HGNC Symbol;Acc:HGNC:29328]	2.175	1.121125	0.037512
U1 spliceosomal RNA [Source:RFAM;Acc:RF00003]	0.237	-2.07852	0.038823
Small nucleolar RNA SNORA63 [Source:RFAM;Acc:RF00092]	0.042	-4.57479	0.039335
LY6/PLAUR domain containing 6 [Source:VGNC Symbol;Acc:VGNC:31103]	5.16	2.367299	0.039923
transgelin [Source:VGNC Symbol;Acc:VGNC:35585]	0.373	-1.42393	0.040281
acid phosphatase 3 [Source:VGNC Symbol;Acc:VGNC:52603]	2.084	1.059579	0.040425
protein phosphatase, Mg2+/Mn2+ dependent 1J [Source:VGNC Symbol;Acc:VGNC:54471]	0.498	-1.00693	0.040509
potassium inwardly rectifying channel subfamily J member 2 [Source:VGNC Symbol;Acc:VGNC:30460]	2.056	1.039925	0.040738
hematopoietic cell-specific Lyn substrate 1 [Source:VGNC Symbol;Acc:VGNC:29775]	11.39	3.509638	0.041062
phosphodiesterase 6G [Source:VGNC Symbol;Acc:VGNC:32683]	0.268	-1.90135	0.041441
G protein subunit gamma 3 [Source:VGNC Symbol;Acc:VGNC:29466]	0.478	-1.06635	0.041895
H2A clustered histone 4 [Source:VGNC Symbol;Acc:VGNC:83550]	0.108	-3.21758	0.041923
glutathione S-transferase theta 2 [Source:NCBI gene (formerly Entrezgene);Acc:516190]	0.17	-2.55855	0.042025
dipeptidase 3 [Source:VGNC Symbol;Acc:VGNC:52194]	0.153	-2.71063	0.042234
BCL2 apoptosis regulator [Source:VGNC Symbol;Acc:VGNC:26445]	2.097	1.068649	0.043013

epsin 3 [Source:VGNC Symbol;Acc:VGNC:28548]	0.493	-1.02077	0.043356
H4 clustered histone 1 [Source:VGNC Symbol;Acc:VGNC:83549]	0.153	-2.70464	0.044024
U1 spliceosomal RNA [Source:RFAM;Acc:RF00003]	0.223	-2.16618	0.045608
adipogenin [Source:VGNC Symbol;Acc:VGNC:25676]	0.076	-3.71487	0.045662
myosin light chain 4 [Source:VGNC Symbol;Acc:VGNC:31802]	0.152	-2.71557	0.046501
glial fibrillary acidic protein [Source:VGNC Symbol;Acc:VGNC:29323]	0.157	-2.66858	0.047185
monocyte to macrophage differentiation associated 2 [Source:VGNC Symbol;Acc:VGNC:31513]	4.4	2.137478	0.047436
ADAM metallopeptidase domain 11 [Source:VGNC Symbol;Acc:VGNC:55768]	0.47	-1.09073	0.047547
UL16-binding protein 17 [Source:NCBI gene (formerly Entrezgene);Acc:100124404]	2.772	1.470937	0.047896
DNA topoisomerase I mitochondrial [Source:HGNC Symbol;Acc:HGNC:29787]	0.492	-1.02415	0.0481
Small nucleolar RNA SNORD15 [Source:RFAM;Acc:RF00067]	0.141	-2.82223	0.048998
FCH and mu domain containing endocytic adaptor 1 [Source:VGNC Symbol;Acc:VGNC:28935]	0.393	-1.3483	0.049137
