# Supplementary material for: The cooperative regulatory effect of the miRNA-130 family on milk fat metabolism in dairy cows
Source: Anim Biosci. 2024 Apr 25;37(7):1289–302. doi: 10.5713/ab.23.0485 (PMC11222843; doi:10.5713/ab.23.0485)
Supplement: Supplementary file 2 [file ab-23-0485-Supplementary-Table-2-4.pdf]

Supplementary table S2: Small RNA sequence

Name	Mature sequence	Mature sequence	sense（5'-3'）	antisense（5'-3'）
>bta-miR-130	CAGUGCAAUGAUGAAAGGGCAU	SiRNA-PPARG	GCCAACACUCAGCUAAGUUTT	AACUUAGCUGAGUGUUGGCTT

Supplementary table S3: Gene primers

**Gene**	**Primer sequence (5 ´to 3 ´)**	**Size(bp)**
DGAT1	CCACTGGGACCTGAGGTGTC	111
	GCATCACCACACACCAATTCA	
FASN	GGGCTCCACCACCGTGTTCCA	226
	GCTCTGCTGGGCCTGCAGCTG	
SCD1	CCATCGCCTGTGGAGTCAC	256
	GTCGGATAAATCTAGCGTAGCA	
ACACA	CTCCAACCTCAACCACTACGG	171
	GGGGAATCACAGAAGCAGCC	
HSL	GGGAGCACTACAAACGCAACG	118
	TGAATGATCCGCTCAAACTCG	
UXT	TGTGGCCCTTGGATATGGTT	101
	GGTTGTCGCTGAGCTCTGTG	
PPARγ	CCTTCACCACCGTTGACTTCT	145
	GATACAGGCTCCACTTTGATTGC	
ATGL	GGAGCTTATCCAGGCCAATG	180
	TGCGGGCAGATGTCACTCT	

Supplementary table S4: Luciferase vector primers

**Primers name**	**Primer sequence 5'-3'**
PPARG -up	ccgctcgagACACGAGCAGCAGAAGCAAG
PPARG -Down	ACGAGAAACAAAGAGGAAGcgccggcgtataaa
PPARG -overlap	CCAACACCCAATTTTTTTTAAAA
